# Survey of rumen microbiota of domestic grazing yak during different growth stages revealed novel maturation patterns of four key microbial groups and their dynamic interactions

**DOI:** 10.1186/s42523-020-00042-8

**Published:** 2020-07-14

**Authors:** Wei Guo, Mi Zhou, Tao Ma, Sisi Bi, Weiwei Wang, Ying Zhang, Xiaodan Huang, Le Luo Guan, Ruijun Long

**Affiliations:** 1 College of Pastoral Agriculture Science and Technology, State Key Laboratory of Grassland Agro-ecosystems, Lanzhou University, Lanzhou, 730020 China; 2 Department of Agricultural, Food and Nutritional Science, University of Alberta, Edmonton, AB T6G 2P5 Canada; 3 Key laboratory of Feed Biotechnology of the Ministry of Agriculture and Rural Affairs, Feed Research Institute, Chinese Academy of Agricultural Sciences, Beijing, 100081 China; 4 School of Life Sciences, Lanzhou University, Lanzhou, 730020 China; 5 School of Public Health, Lanzhou University, Lanzhou, 730020 China; 6 International Centre for Tibetan Plateau Ecosystem Management, Lanzhou University, Lanzhou, 730020 China

**Keywords:** Domestic grazing yaks, Rumen microbiota, Age-discriminatory taxa, Maturation, Keystone species, Dynamic interactions

## Abstract

**Background:**

The development and maturation of rumen microbiota across the lifetime of grazing yaks remain unexplored due to the varied lifestyles and feed types of yaks as well as the challenges of obtaining samples. In addition, the interactions among four different rumen microbial groups (bacteria, archaea, fungi and protozoa) in the rumen of yak are not well defined. In this study, the rumen microbiota of full-grazing yaks aged 7 days to 12 years old was assessed to determine the maturation patterns of these four microbial groups and the dynamic interactions among them during different growth stages.

**Results:**

The rumen microbial groups (bacteria, archaea, protozoa and fungi) varied through the growth of yaks from neonatal (7 days) to adult (12 years), and the bacterial and archaeal groups were more sensitive to changes in growth stages compared to the two eukaryotic microbial groups. The age-discriminatory taxa within each microbial group were identified with the random forest model. Among them, *Olsenella* (bacteria), Group 10 sp., belonging to the family *Methanomassiliicoccaceae* (archaea), *Orpinomyces* (fungi), and *Dasytricha* (protozoa) contributed the most to discriminating the age of the rumen microbiota. Moreover, we found that the rumen archaea reached full maturation at 5 approximately years of age, and the other microbial groups matured between 5 and 8 years of age. The intra-interactions patterns and keystone species within each microbial group were identified by network analysis, and the inter-interactions among the four microbial groups changed with growth stage. Regarding the inter-interactions among the four microbial groups, taxa from bacteria and protozoa, including *Christensenellaceae* R-7 group*, Prevotella* 1*, Trichostomatia*, *Ruminococcaceae* UCG-014 and *Lachnospiraceae*, were the keystone species in the network based on betweenness centrality scores.

**Conclusions:**

This study depicted a comprehensive view of rumen microbiota changes in different growth stages of grazing yaks. The results revealed the unique microbiota maturation trajectory and the intra- and inter-interactions among bacteria, archaea, fungi and protozoa in the rumen of grazing yaks across the lifetime of yaks. The information obtained in this study is vital for the future development of strategies to manipulate rumen microbiota in grazing yaks for better growth and performance in the harsh Qinghai-Tibetan Plateau ecosystem.

## Background

The yak (*Bos grunniens*), inhabiting the Qinghai-Tibetan Plateau in China, diverged from other ruminant animals millions of years ago [1]. The Qinghai-Tibetan Plateau is the world’s highest plateau with altitudes ranging from 4000 to 5500 m [2], featuring an extreme environment with low ambient temperature and partial pressure of oxygen, as well as a high level of ultraviolet radiation [3]. Yak has developed many anatomical and physiological traits to adapt to this extreme living habitat, including large lungs and hearts, increased foraging ability, high energy metabolism, and lack of hypoxic pulmonary vasoconstriction [4, 5]. Traditionally, yaks graze yearly on native pastures with coarse grasses as their only food source [6]. Although they suffer from inadequate feed and malnutrition in the long and cold seasons [7], they degrade lignocellulose better and have more efficient energy (producing more short chain fatty acids in the rumen) and nitrogen metabolism (higher nitrogen retention) compared to cattle under the same conditions [8, 9].

Similar to other ruminants, the rumen of yak contains complex symbiotic microorganisms that ferment fibrous plant materials, providing the host with usable nutrients such as volatile fatty acids (VFAs) and microbial proteins [10]. A recent study revealed that the proportion of uncultured microbial species was higher in the rumen of naturally grazing yaks than in the rumen of house-farmed Jinnan cattle fed diets with similar energy densities or with high-energy diets [11], and yaks had a unique rumen archaeal community in comparison to domestic cattle under the same grazing conditions [12]. Moreover, it has been reported that individuality [13], feed type [14] and feeding regimes [15, 16] can affect bacterial and archaeal composition in the rumen of yak. Recent research based on rumen metagenomes and host transcriptomes revealed that the rumen of yak contained fewer microbial genes involved in methane emission and a higher abundance of genes involved in host VFA absorption compared with its low-altitude relatives (cattle), suggesting that rumen microbiota co-evolved with the host genome to adapt to extreme environmental conditions [17]. However, most studies mentioned above have only focused on bacterial and archaeal domains and one sampling timepoint. It is known that eukaryotic microbes in the rumen also play important roles in rumen fermentation. Rumen fungi play a significant role in lignocellulosic material degradation, providing a source of fermentable sugars for other microbes and the host [18]. Indeed, it has been reported that the abundance of rumen protozoa was positively associated with total VFA production and the molar proportion of butyrate in the rumen of lambs [19]. Thus, we comprehensively investigated rumen microbial composition (including bacteria, archaea, protozoa and fungi) and their interactions with each other, which is essential for developing appropriate manipulation strategies that promote yaks to better adapt to the extreme grazing environment.

Increasing evidence has highlighted the importance of early-life microbial colonization and its impact on lifelong animal productivity and health [20–27]. It has been shown that the rumen bacterial community is established before the intake of solid food and its composition changes with age [20, 21], and the rumen microbiota (bacteria, archaea and fungi) exhibits an adult-like microbiota between weaning and 1 year of age in dairy cows [22]. In addition, studies have reported that rumen bacterial and archaeal communities continue to evolve and mature with aging even after adulthood [23, 24]. Studies on early-life interventions of rumen microbial community colonization have found that different diets affect microbiota (bacteria, archaea and fungi) development in calves, and some calf-diet-driven differences are apparent in the microbiota of adult cows [25, 26]. For example, feeding a methanogen inhibitor (bromochloromethane) to goat kids affected rumen archaeal community colonization, and the effect of this inhibitor on some less abundant archaeal groups persisted 4 months after exposure [27]. These findings suggest that early-life interventions could lead to assembly of a specific composition or promote the development of rumen that potentially persists later in life, affecting health and productivity [28]. However, there is a paucity of knowledge of how rumen microbiota develops across different growth stages and when it becomes fully maturated over the lifetime of grazing yaks. Therefore, in the present study, we assessed the rumen microbiota of grazing yaks as a whole, including bacteria, archaea, protozoa and fungi, from 7 days to 12 years of age using amplicon sequencing, with the aim of addressing the above knowledge gaps. In addition, the dynamic interactions within and among different microbial groups under different growth stages were also explored to identify whether the compositional changes at each microbial group level could affect the microbial-microbial interactions. Understanding the phylogenetic composition, interactions and maturation of the whole rumen microbiota across the lifetime of yaks will provide the fundamental knowledge needed for developing strategies to improve rumen function in the extreme Qinghai-Tibetan Plateau environment.

## Results

### Diversity of rumen microbiota of grazing yaks in different growth stages

**Fig. 1:**
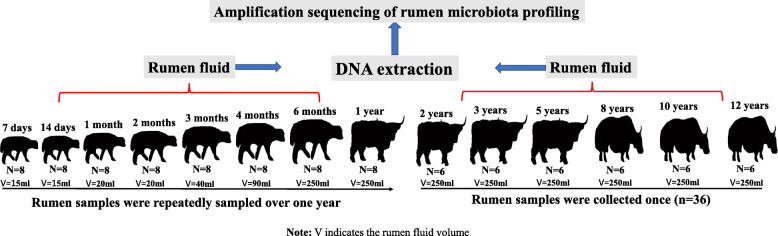
Experimental design for rumen sample collection

Amplicon sequencing of bacteria, archaea, protozoa, and fungi in 100 rumen samples collected from yaks aged 7 days to 12 years old across 14 time points (Fig. 1) was performed; however, high-quality sequence data were obtained for 80, 75, 71, and 65 samples, for bacteria, archaea, protozoa, and fungi, respectively. Detailed information on the number of samples is listed in Table 1. Here, 7 days to 1 year of age was considered the pre-weaned stage; 2 to 3 years of age was considered the youth stage (puberty); 5 to 12 years of age was considered the adult stage based on a previous description [29].

**Table 1:** Sample information and sequencing statistics obtained using the DADA2 algorithm in QIIME2

Age group	Number of samples	Number of sequences	Frequency	Number of ESVs	Good's coverage
Bacteria					
7d	5	52,238 ± 12,630	30,010 ± 12,440	319 ± 120	99.32% ± 0.003
14d	5	55,016 ± 18,393	16,227 ± 5805	291 ± 144	99.57% ± 0.003
1m	6	60,430 ± 12,638	27,747 ± 7213	394 ± 175	99.23% ± 0.004
2m	5	52,145 ± 16,144	15,312 ± 4997	697 ± 144	98.90% ± 0.005
3m	6	56,182 ± 16,390	15,824 ± 5856	738 ± 263	98.68% ± 0.008
4m	7	56,350 ± 12,375	16,736 ± 4395	810 ± 193	98.66% ± 0.005
6m	6	59,274 ± 4492	14,909 ± 2458	848 ± 80	98.67% ± 0.003
1y	5	56,981 ± 16,552	14,641 ± 6165	883 ± 234	98.44% ± 0.006
2y	6	56,913 ± 6502	14,646 ± 2829	994 ± 172	98.31% ± 0.005
3y	5	55,345 ± 11,704	13,502 ± 4249	988 ± 316	98.25% ± 0.009
5y	6	50,667 ± 9955	10,604 ± 3126	835 ± 214	98.72% ± 0.005
8y	6	55,476 ± 15,398	12,290 ± 4057	962 ± 277	98.35% ± 0.007
10y	6	60,056 ± 7647	13,461 ± 1935	1074 ± 139	98.14% ± 0.003
12y	6	55,415 ± 12,325	12,414 ± 3633	995 ± 281	98.25% ± 0.006
*P* value				< 0.0001	> 0.05
Archaea					
14d	5	19,372 ± 3328	10,754 ± 3343	44 ± 12	99.94% ± 0.0001
1m	5	16,897 ± 4360	10,402 ± 2831	40 ± 23	99.90% ± 0.0001
2m	5	19,169 ± 6558	9066 ± 2217	28 ± 8	99.99% ± 0.0001
3m	6	14,920 ± 2467	6541 ± 1574	27 ± 7	99.99% ± .0001
4m	7	16,856 ± 3321	7817 ± 1699	38 ± 10	99.98% ± 0.0002
6m	6	13,792 ± 4255	6179 ± 1999	38 ± 5	99.99% ± 0.0001
1y	5	18,556 ± 5254	8282 ± 2695	43 ± 5	99.99% ± 0.00003
2y	6	15,102 ± 5243	7667 ± 2777	47 ± 11	99.97% ± 0.0002
3y	6	16,690 ± 4738	7392 ± 1563	46 ± 4	99.99% ± 0.0001
5y	6	15,866 ± 2104	6553 ± 1827	42 ± 7	99.99% ± 0.0001
8y	6	15,490 ± 994	7309 ± 816	47 ± 3	99.99% ± 0.0001
10y	6	15,643 ± 4837	6672 ± 11	43 ± 11	99.99% ± 0.0002
12y	6	19,596 ± 3692	7472 ± 1577	42 ± 5	99.99% ± 0.0001
*P* value				0.007	> 0.05
Fungi					
1m	5	63,933 ± 12,105	43,217 ± 17,325	26 ± 11	99.79% ± 0.0003
2m	5	63,156 ± 8927	43,383 ± 13,052	49 ± 18	99.95% ± 0.0004
3m	5	65,614 ± 13,940	42,452 ± 9451	48 ± 16	99.97% ± 0.0001
4m	6	67,611 ± 7412	47,666 ± 7239	46 ± 17	99.96% ± 0.0001
6m	5	69,635 ± 4601	31,080 ± 23,236	41 ± 22	99.95% ± 0.0006
1y	5	63,303 ± 6821	42,651 ± 4103	43 ± 18	99.96% ± 0.0003
2y	6	70,881 ± 4236	51,483 ± 2749	57 ± 18	99.94% ± 0.0003
3y	6	64,915 ± 9110	44,740 ± 6529	66 ± 12	99.95% ± 0.0002
5y	5	59,552 ± 16,489	39,604 ± 9599	52 ± 22	99.97% ± 0.0002
8y	6	66,924 ± 3850	45,155 ± 3932	68 ± 25	99.94% ± 0.0006
10y	5	70,885 ± 1638	48,606 ± 2227	70 ± 12	99.93% ± 0.0004
12y	6	66,724 ± 8008	39,244 ± 6064	65 ± 15	99.96% ± 0.0001
*P* value				0.008	> 0.05
Protozoa					
1m	6	22,409 ± 7699	15,927 ± 5932	31 ± 16	99.86% ± 0.0007
2m	5	18,364 ± 1862	12,538 ± 2959	12 ± 5	99.93% ± 0.0003
3m	6	16,334 ± 3107	7184 ± 3070	20 ± 8	99.92% ± 0.0011
4m	7	17,212 ± 4875	7376 ± 3130	24 ± 13	99.91% ± 0.0008
6m	6	20,446 ± 5872	6143 ± 1896	41 ± 15	99.75% ± 0.0017
1y	5	19,728 ± 1913	6910 ± 1912	48 ± 7	99.81% ± 0.0005
2y	6	18,919 ± 6117	7068 ± 2740	65 ± 27	99.51% ± 0.0026
3y	6	15,910 ± 3081	5687 ± 1563	73 ± 11	99.54% ± 0.0009
5y	6	16,860 ± 3405	5547 ± 1405	68 ± 11	99.48% ± 0.0001
8y	6	19,072 ± 7301	6817 ± 3726	91 ± 20	99.36% ± 0.0031
10y	6	16,736 ± 2231	5934 ± 1709	72 ± 11	99.51% ± 0.0010
12y	6	17,061 ± 2908	6826 ± 2536	73 ± 16	99.60% ± 0.0011
*P* value				< 0.0001	> 0.05

**Fig. 2:**
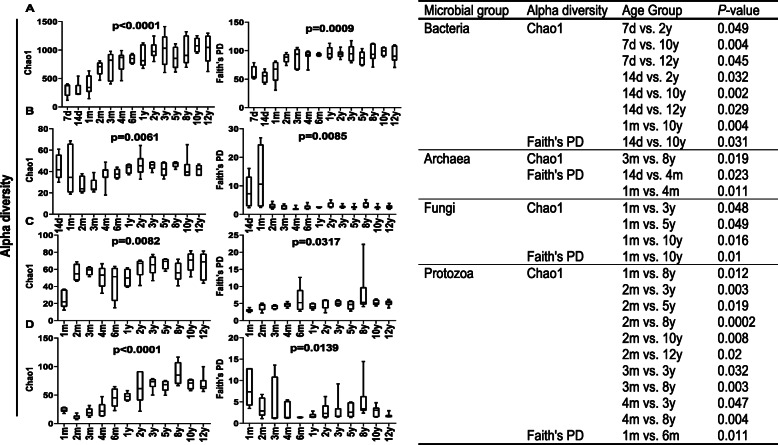
Chao1 index and Faith’s phylogenetic diversity of rumen bacteria (**a**), archaea (**b**), fungi (**c**) and protozoa (**d**). The table on the right shows the statistical significance

Bacteria were detected at 7 days of age, archaea were detected at 14 days of age, and fungi and protozoa were both detected at 1 month of age (Table 1). For bacteria, 1,296,989 quality-controlled reads were generated, with a mean ± SD of 16,212 (± 7253) sequences per sample, and 26,738 unique exact sequence variants (ESVs) were identified (ranging from 123 to 1437 per sample) (Table 1). Similarly, a total of 581,950 (7759 ± 2364 per sample), 2,824,701 (43,457 ± 10,633 per sample), and 551,668 (7770 ± 4054 per sample) high-quality reads were obtained for archaea, fungi and protozoa, yielding 359 (ranging from 18 to 68), 3230 (ranging from 13 to 115), and 387 unique ESVs (ranging from 7 to 124), respectively. Based on the sampling depth at which rarefaction curves tend to plateau, we rarefied each sample to 7158 (bacteria), 3794 (archaea), 4761 (fungi) and 1818 (protozoa) reads (Additional file 1: Figure S1). The Good’s coverage index was 98.7% (± 0.7%) for bacteria, 99.9% (± 0.1%) for archaea, 99.9% (± 0.03%) for fungi, and 99.7% (± 0.2%) for protozoa (Table 1), indicating that the sequencing depth was adequate to represent each rumen microbial community. To gain insight into the diversity of the rumen microbiota, we compared the Chao1 and Faith’s phylogenetic diversity (PD) indices across age groups. The Chao1 index of the rumen bacteria increased with age up to 2 years old and then remained stable (Fig. 2a), while those of rumen archaea and protozoa were similar up to 2 months, generally increased until the age of 2 years and remained stable afterwards (Fig. 2b and d). However, the Chao1 index of fungi increased from 1 month to 3 months, decreased by the age of 2 years, and further increased with age (Fig. 2c). The PD index of the rumen bacteria and fungi (*P* < 0.05) significantly increased with age, whereas that of the rumen archaea and protozoa decreased with age, although there were some fluctuations (Fig. 2, Additional file 2: Table S1).

### Microbial profiles of rumen microbiota in grazing yaks

**Fig. 3:**
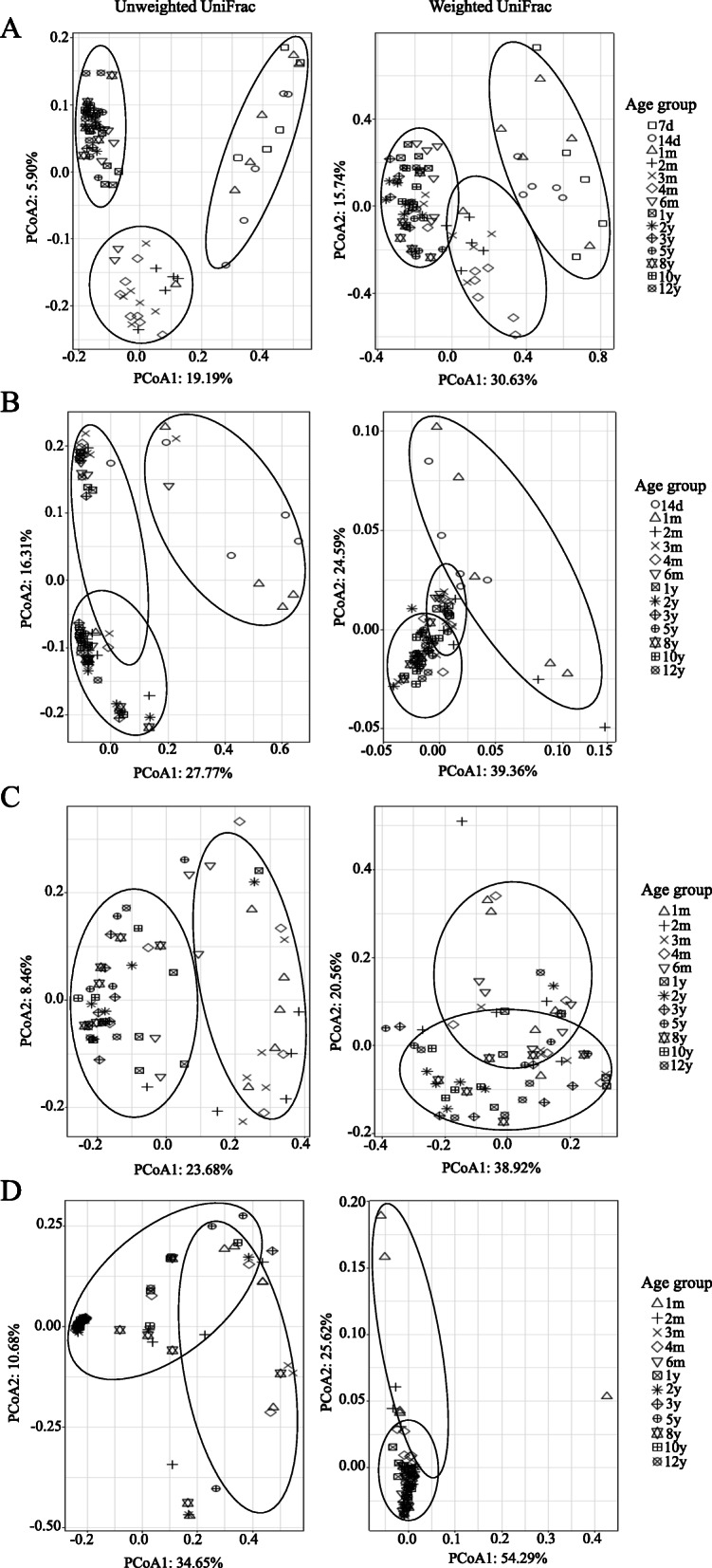
Principal coordinate analysis (PCoA) based on weighted and unweighted UniFrac distances. Changes in the rumen bacterial (**a**), archaeal (**b**), fungal (**c**) and protozoal (**d**) community structure with growth stage

Next, principal coordinates analysis (PCoA) based on unweighted and weighted UniFrac distance matrices was performed to determine whether the microbial community structure changed with increasing age. The PCoA plot showed clear age-based separation of rumen bacteria between 7 days to 1 month, 2–4 months, and 6 months to 12 years of age (Fig. 3a). Similarly, three clusters were formed for rumen archaea, where 14 days to 1 month formed one cluster, 2 months to 1 year formed another cluster, and the remaining age groups (2–12 years) formed another cluster (Fig. 3b). For fungi, we observed clear separations between 1 month to 1 year and 2 to 12 years of age (Fig. 3c). However, no clear separation was found for rumen protozoa (Fig. 3d).

In addition, the permutational analysis of variance (PERMANOVA) showed that profiles of bacteria, archaea and fungi significantly differed among different age groups (Benjamini-Hochberg corrected *P* < 0.05, Additional file 2: Table S2). For protozoa, it was especially significant between young age groups (1 to 4 months of age) and old age groups (3 to 12 years of age) (Benjamini-Hochberg corrected *P* < 0.01). The UniFrac dissimilarity of the bacterial community decreased with age (*P* < 0.01), as the distance between samples decreased with increasing age (Additional file 3: Figure S2A). The highest dissimilarity of the archaeal community was observed at 1 month of age and then decreased with age (*P* < 0.01, Additional file 3: Figure S2B). However, the decrease in dissimilarity with the increase in age was less apparent for rumen fungi and protozoa (*P* < 0.05, Additional file 3: Figure S2C and 2D), except for the weighted UniFrac dissimilarity in the fungal community (Additional file 3: Figure S2C).

### Shifts in rumen microbiota at different taxonomic levels

#### Changes in the rumen bacterial composition with yak growth

For bacteria, sequence variants were classified into 368 genera belonging to 26 phyla. At the phylum level, 16 phyla were referred to as the detected bacterial phyla (relative abundance > 0.1% and present in more than half of the total animal populations per age group). The most abundant phylum was *Bacteroidetes* (41.7 to 74.1%), followed by *Firmicutes* (18.2 to 41.7%), *Proteobacteria* (0.6 to 11.3%), *Patescibacteria* (0.03 to 8.9%), *Actinobacteria* (0.2 to 2.9%) and *Planctomycetes* (0.01 to 3.1%) (Additional file 4: Figure S3A). The relative abundances of five phyla, *Proteobacteria*, *Spirochaetes*, *Kiritimatiellaeota*, *Actinobacteria*, and *Patescibacteria*, were significantly different among age groups based on DESeq2 analysis (*P* < 0.05, Additional file 2: Table S3). At the genus level, 64 genera were considered detectable using the same cut-offs mentioned above (Additional file 5: Figure S4A). Among them, *Prevotella* 1 (13.1 ± 0.1%) and *Rikenellaceae* RC9 gut group (10.8 ± 0.1%) were predominant regardless of age. Further differential abundance analysis using “DESq2” [30] identified 38 genera that were significantly different among age groups (Additional file 2: Table S3).

#### Changes in the rumen archaeal composition with yak growth

Taxonomic analysis revealed that most ESVs, from 94.4 to 99.8%, belonged to the phylum *Euryarchaeota* (Additional file 4: Figure S3B). At the species level, 35 species were identified, and 10 of them were considered detectable (relative abundance > 0.1% and present in more than half of the total animal populations per age group, Additional file 5: Figure S4B). Among these species, the *Methanobrevibacter ruminantium* clade (37.8 ± 10.2%), the *Methanobrevibacter gottschalkii* clade (34.8 ± 0.1%), *Methanobacterium alkaliphilum* (10.2 ± 0.1%), and Group 10 sp. belonged to the family *Methanomassiliicoccaceae* (5.9 ± 0.04%); *Methanosphaera* sp. ISO3-F5 (5.0 ± 0.02%) was the top five most abundant species, accounting for 93.7 ± 0.1% of the total number of sequences. Differential abundance analysis using DESeq2 showed that the relative abundances of the *Methanobrevibacter ruminantium* clade (55.8 ± 0.2%), Group 10 sp. (1.2 ± 0.01%) and Group 12 sp. ISO4 − H5 (0.1 ± 0.0%) were significantly different among age groups (*P* < 0.05, Additional file 2: Table S3). Specifically, ESVs belonging to the species of the *Methanobrevibacter ruminantium* clade were predominant at 1 month of age, while ESVs belonging to species Group 10 sp. belonged to the family *Methanomassiliicoccaceae*, and *Methanosphaera* sp. ISO3-F5 was less abundant at 1 year and 5 years of age, respectively.

#### Changes in the rumen fungal composition with yak growth

The fungal phylum *Neocallimastigomycota* (99.9 ± 0.3%, Additional file 4: Figure S3C) was the most dominant irrespective of age. In total, 7 fungal taxa were identified at the genus level, and 6 were considered detectable according to the selected criteria mentioned above (Additional file 5: Figure S4C). Among them, the top three genera were *Caecomyces* (35.3 ± 0.3%), *Orpinomyces* (29.1 ± 0.3%) and *Neocallimastix* (4.8 ± 0.1%). No ESVs were differentially abundant among age groups.

#### Changes in the rumen protozoal composition with yak growth

Four protozoal phyla were identified, of which the most abundant phylum was SAR, with a relative abundance of 99.4% (± 0.02) (Additional file 4: Figure S3D). In this phylum, the most abundant ESVs were classified as subclass *Trichostomatia* (40.4 ± 0.3%). At the genus level, a total of 11 genera were identified, and 7 genera were detectable, with *Entodinium* (19.2 ± 0.2%), *Dasytricha* (17.2 ± 0.2%) and *Diplodinium* (9.2 ± 0.2%) being the most abundant (Additional file 5: Figure S4D). There were no ESVs that differed significantly among age groups.

### Maturation patterns differed among different microbial groups

**Fig. 4:**
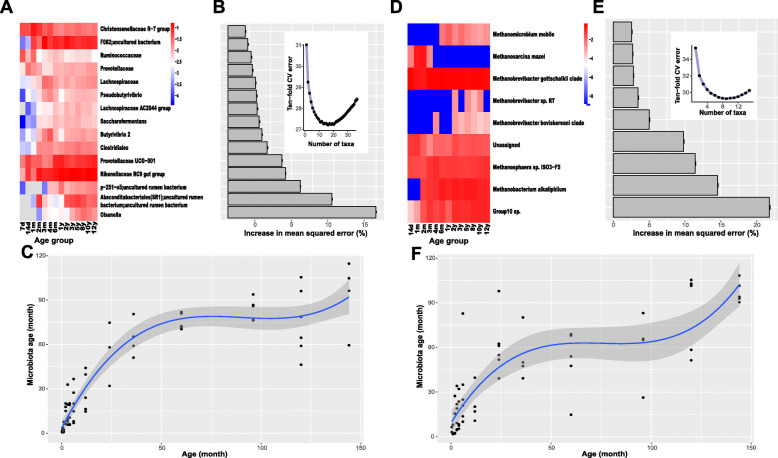
Bacterial and archaeal taxonomic biomarkers for defining rumen microbiota maturation in grazing yaks. **a, d** Heatmap of the mean relative abundance of the age-discriminatory taxa against time of sampling for each age group. **b, e** Age-discriminatory taxa were identified by applying random forest regression of the relative abundances of taxa in rumen fluid against chronological age of grazing yaks. Importance was determined based on the percentage increase in the mean-squared error of the predicted microbiota age when the relative abundance values of each taxon were randomly permuted (mean importance ± sem., n = 100 replicates). The inset shows 10-fold cross-validation error as a function of the number of input taxa used to regress against the age of yaks in the training set, in order of variable importance. **c, f** Microbiota age predictions plotted against chronological age. The curve is a smoothed spline fit between microbiota age and chronological age

**Fig. 5:**
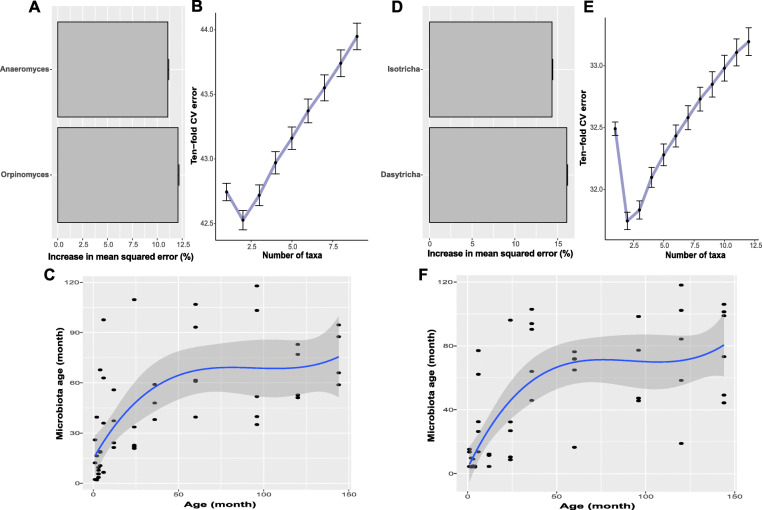
Fungal and protozoal taxonomic biomarkers for defining rumen microbiota maturation in grazing yaks. **a, d** Age-discriminatory taxa were identified by applying random forest regression of the relative abundances of taxa in rumen fluid against chronological age of grazing yaks. Importance was determined based on the percentage increase in the mean-squared error of the predicted microbiota age when the relative abundance values of each taxon were randomly permuted (mean importance ± sem., *n* = 100 replicates). **b, e** The 10-fold cross-validation error as a function of the number of input taxa used to regress against the age of yaks in the training set, in order of variable importance. **c, f** Microbiota age predictions plotted against chronological age. The curve is a smoothed spline fit between microbiota age and chronological age

The random forest regression model [31] was used to identify the age-discriminatory taxa of the rumen microbiota. In total, 15 bacterial (Fig. 4a), 9 archaeal (Fig. 4d), 2 protozoal (Fig. 5d) and 2 fungal (Fig. 5a) age-discriminatory taxa were identified according to the feature importance scores and 10-fold cross-validation (Additional file 2: Table S4). Specifically, the bacterial genus *Olsenella* (0.8 ± 0.01%) contributed the most to discriminating yak rumen microbiota differences according to age (Fig. 4a), while for the other three microbial groups, Group 10 sp. (6.1 ± 0.1%, archaea), *Orpinomyces* (22.9 ± 0.3%, fungi) and *Dasytricha* (17.2 ± 0.2%, protozoa) contributed the most to discriminating yak rumen microbiota differences according to age (Fig. 4d, Fig. 5a and d).

The regression model explained 70–72%, 45–54%, 23–25% and 48–50% of the variance in rumen bacteria, archaea, fungi and protozoa, respectively, based on the above age-discriminatory taxa. There was little improvement in the predictive performance (estimated by 10-fold cross validation) when any additional taxa beyond the age-discriminatory taxa were added for all four microbial groups (Fig. 4b and e; Fig. 5b and e). To quantitatively identify when the rumen microbiota mature, a smoothing spline function was fit between microbiota age and the corresponding chronological age of the animals. As a result, we found that the rumen bacteria reached full maturation between 5 and 8 years of age and archaea reached full maturation at approximately 5 years of age as the smoothed spline fit curve was saturated during this period (Fig. 4c and f). Similar to bacteria, fungi and protozoa reached full maturation between 5 and 8 years of age (Fig. 5c and f).

### Intra- and inter-group microbial interactions in the rumen of grazing yaks

**Fig. 6:**
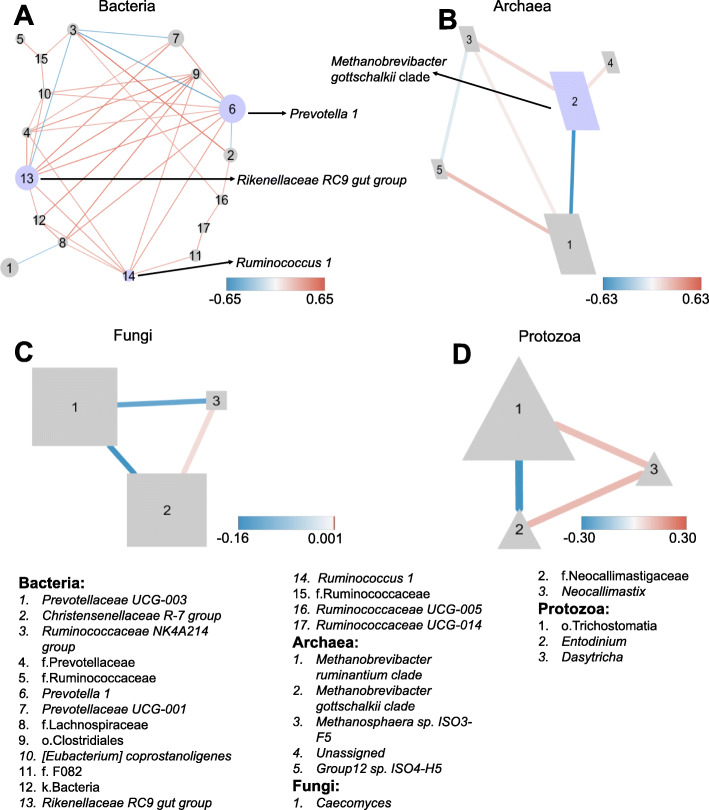
Co-occurring network analysis of intra-interactions of core taxa in each rumen microbial kingdom. The association patterns of bacteria (**a**), archaea (**b**), fungi (**c**) and protozoa (**d**) from birth to adulthood. The size of each node is proportional to the relative abundance. The lines in red and blue denote positive and negative correlations, respectively. The nodes are referred to as keystone species and are highlighted in light purple, and the taxonomic names of keystone species are indicated in the networks. The taxonomic information of the nodes is shown at the bottom of the figures

We first employed a co-occurrence network based on correlation relationships and *P*-values adjusted with FDR (false discovery rate) to explore the intragroup interactions of core taxa identified in all age groups (> 50% of the population of each age group). For bacteria, there were 17 nodes and 38 edges, with most nodes belonging to the phyla *Bacteroidetes* and *Firmicutes* (Fig. 6a). The topological properties were calculated to describe the complexity of the network (Additional file 2: Table S5). The top three genera identified as keystone taxa were *Prevotella* 1, *Ruminococcaceae* NK4A214 group and *Ruminococcus* 1 based on betweenness centrality scores (Fig. 6a), which measures the number of shortest paths going through a given node, as a proxy for the location of this node in relation to other nodes [32]. For archaea, the resulting co-occurrence network consisted of 5 nodes and 12 edges (Fig. 6b), and the *Methanobrevibacter gottschalkii* clade was identified as a keystone taxon based on its betweenness centrality score in the network (Additional file 2: Table S5). For fungi and protozoa, the network was less complicated. In the fungal network, *Caecomyces* was negatively correlated with the rest of the nodes (*Neocallimastix* and *Neocallimastigaceae*) that had a positive correlation (Fig. 6c). In the protozoal network, *Dasytricha* had a positive correlation with the other nodes (*Entodinium* and *Trichostomatia*) that had a positive correlation (Fig. 6d).

Next, we performed a meta-community co-occurrence network to explore the inter-group interactions of core taxa (please see methods). This generated a meta-community network with 46 links from 24 nodes (Additional file 6: Figure S5), including 17 bacterial nodes, 3 archaeal nodes, 1 fungal node, and 3 protozoal nodes. The relative importance of individual nodes within the network was computed via the topological features (Additional file 2: Table S6). Based on betweenness centrality scores, the top five taxa regarded as keystone species (Additional file 6: Figure S5) were *Christensenellaceae* R-7 group (3.9 ± 0.04%), *Prevotella* 1 (13.3 ± 0.1%), *Trichostomatia* (40.4 ± 0.3%), *Ruminococcaceae* UCG-014 (0.5 ± 0.02%) and *Lachnospiraceae* (1.2 ± 0.01%).

The intra- and inter-group interactions of core taxa under each age group (age-specific taxa) were further explored using co-occurrence network analysis. We first investigated the intra-interactions of age-specific taxa and found that the network complexity (as determined by the clustering coefficient and average degree scores) in rumen archaea, fungi and protozoa varied greatly with age, while that in bacteria changed at the first growth stages and remained stable after 3 years of age (Table 2). In addition, we identified 5 and 3 keystone species for bacteria and archaea, respectively, based on the betweenness centrality score. For bacteria, the keystone species varied with age, and only a few taxa were shared by some age groups. For example, *Bacteroidales* RF16 group uncultured bacterium (shared in 3 months, 6 months, 2 years and 10 years of age) and *Streptococcus* (shared in month 1, month 3 and 5 years of age) (Additional file 2: Table S7). For archaea*,* although the keystone species (top 3) differed among each age group, some taxa were shared by multiple age groups, including the *Methanobrevibacter ruminantium* clade and *Methanobrevibacter gottschalkii* clade (Additional file 2: Table S7). Similar to bacteria and archaea, the keystone species of fungi and protozoa changed with age, and some were shared by many age groups (Additional file 2: Table S7). Specifically, the fungi *Caecomyces*, *Anaeromyces*, *Orpinomyces*, and *Neocallimastigaceae* were shared by at least three age groups, while the protozoa *Entodinium*, *Trichostomatia* and *Trichostomatia* uncultured were shared by at least four age groups (Additional file 2: Table S7). Next, we examined the inter-kingdom interactions between age-specific taxa and found that the complexity of networks fluctuated greatly at the early growth stages (1 month to 2 years); the majority of keystone species (top 5) were from bacteria in all age groups, and unique keystone species were found in each age group (Table 3).

**Table 2:** Global topological properties of co-occurring networks of intra-interactions in each microbial kingdom

Age group	Number of nodes	Number of edges	Average path length	Graph density	Clustering coefficient	Average degree
Bacteria						
7d	79	1294	1.58	0.42	0.66	32.76
14d	78	1753	1.42	0.58	0.76	44.95
1m	100	1670	1.67	0.34	0.61	33.40
2m	120	3059	1.57	0.43	0.62	50.98
3m	129	2612	1.69	0.32	0.54	40.50
4m	145	2680	1.75	0.26	0.48	36.97
6m	115	2370	1.64	0.36	0.59	41.22
1y	116	3038	1.54	0.46	0.63	52.38
2y	115	2129	1.68	0.32	0.54	37.03
3y	104	2671	1.50	0.50	0.68	51.37
5y	97	1432	1.70	0.31	0.52	29.53
8y	113	2075	1.68	0.33	0.54	36.73
10y	118	2102	1.70	0.30	0.50	35.63
12y	109	1942	1.67	0.33	0.58	35.63
Archaea						
14d	7	14	1.33	0.67	0.61	4.00
1m	6	7	1.87	0.47	0.55	2.33
2m	8	14	1.79	0.50	0.85	3.50
3m	8	12	1.08	0.43	0.97	3.00
4m	6	4	1.57	0.27	0	1.33
6m	8	15	1.54	0.54	0.72	3.75
1y	9	16	1.69	0.44	0.58	3.56
2y	10	15	2.27	0.33	0.50	3.00
3y	9	15	1.83	0.42	0.56	3.33
5y	10	17	1.84	0.38	0.52	3.40
8y	9	15	2.06	0.42	0.80	3.33
10y	7	8	2.14	0.38	0.46	2.29
12y	8	22	1.21	0.79	0.78	5.50
Fungi						
1m	2	1	NA	NA	NA	1
2m	5	10	1.00	1.00	1.00	4.00
3m	6	7	1.67	0.47	0.50	2.33
4m	5	5	1.70	0.50	0.50	2.00
6m	6	11	1.27	0.73	0.60	3.67
1y	6	6	1.00	0.40	1.00	2.00
2y	6	9	1.53	0.60	0.71	3.00
3y	8	20	1.29	0.71	0.85	5.00
5y	6	12	1.20	0.80	0.85	4.00
8y	9	27	1.25	0.75	0.78	6.00
10y	7	7	1.36	0.33	0.60	2.00
12y	6	4	1.57	0.27	0	1.33
Protozoa						
1m	4	6	1.00	1.00	1.00	3.00
2m	2	1	NA	NA	NA	1.00
3m	2	1	NA	NA	NA	1.00
4m	6	5	1.17	0.33	0.75	1.67
6m	8	9	2.43	0.32	0.46	2.25
1y	8	12	1.82	0.43	0.58	3.00
2y	8	10	2.18	0.36	0.45	2.50
3y	9	16	1.92	0.44	0.75	3.56
5y	8	22	1.21	0.79	0.81	5.50
8y	10	30	1.36	0.67	0.77	6.00
10y	9	9	1.77	0.25	0.25	2.00
12y	6	10	1.40	0.67	0.69	3.33

**Table 3:** Topological properties and keystone species of co-occurring networks of inter-kingdom interactions

Age group	Clustering coefficient	Average degree	Betweenness centrality	Keystone species	Microbial group
1m	0.67	9.33	389.38	*Bergeyella*	Bacteria
			363.59	*Blautia*	Bacteria
			354.98	*Lachnoclostridium*	Bacteria
			308.46	*Intestinimonas*	Bacteria
			302.13	*Chryseobacterium*	Bacteria
2m	0.60	16.09	421.33	o.Gastranaerophilales	Bacteria
			406.28	*Ruminiclostridium* 6	Bacteria
			391.14	f.Prevotellaceae	Bacteria
			311.28	f.Eggerthellaceae	Bacteria
			279.19	*Veillonellaceae* UCG-001	Bacteria
3m	0.58	11.53	561.04	*Rhodococcus*	Bacteria
			495.59	o.Rhodospirillales	Bacteria
			467.14	*Corynebacterium* 1	Bacteria
			409.25	p-1088-a5 gut group	Bacteria
			385.13	o.Gastranaerophilales	Bacteria
4m	0.55	8.65	555.67	p-251-o5	Bacteria
			535.64	*Anaeromyces*	Fungi
			504.04	f.Absconditabacteriales (SR1)	Bacteria
			468.45	f.F082	Bacteria
			433.09	*Rikenellaceae* RC9 gut group	Bacteria
6m	0.55	12.29	557.57	o.Absconditabacteriales (SR1)	Bacteria
			365.58	[*Eubacterium] nodatum* group	Bacteria
			353.64	f.Marinilabiliaceae	Bacteria
			337.55	*Ophryoscolex*	Protozoa
			297.75	o.Bacteroidales	Bacteria
1y	0.56	15.54	493.14	k.Archaea	Archaea
			357.79	*Anaerovorax*	Bacteria
			357.79	Group9 sp. ISO4-G1	Archaea
			345.73	o.Gastranaerophilales	Bacteria
			345.73	f.Bacteroidales RF16 group	Bacteria
2y	0.50	7.58	390.93	f.Bacteroidales RF16 group	Bacteria
			364.39	*Neocallimastix*	Fungi
			344.68	o.Rickettsiales	Bacteria
			340.19	*Prevotellaceae* UCG-001	Bacteria
			315.33	o.Bacteroidales	Bacteria
3y	0.59	16.72	399.02	o.WCHB1–41	Bacteria
			387.99	*Ruminiclostridium* 1	Bacteria
			365.52	*Solobacterium*	Bacteria
			365.52	f.Muribaculaceae	Bacteria
			364.01	f.Paludibacteraceae	Bacteria
5y	0.53	10.19	371.35	*Oontomyces*	Fungi
			344.05	*candidate division CPR1 bacterium* ADurb.Bin160	Bacteria
			338.71	*Prevotellaceae* YAB2003 group	Bacteria
			318.16	*Erysipelotrichaceae* UCG-004	Bacteria
			296.96	f.Bacteroidales UCG-001	Bacteria
8y	0.53	10.41	455.85	c.Deltaproteobacteria	Bacteria
			442.53	o.Rickettsiales	Bacteria
			362.40	f.F082	Bacteria
			342.17	f.vadinBE97;Ambiguous_taxa	Bacteria
			339.35	*Fibrobacter*	Bacteria
10y	0.52	11.80	526.05	f.Bacteroidales BS11 gut group;Ambiguous_taxa	Bacteria
			496.54	f.Muribaculaceae	Bacteria
			406.49	*Ruminococcaceae* NK4A214 group	Bacteria
			382.65	*Horsej-*a03	Bacteria
			332.04	o.Rickettsiales	Bacteria
12y	0.59	10.54	602.73	f.Bacteroidales RF16 group	Bacteria
			528.41	*candidate division* CPR1 *bacterium*	Bacteria
			514.56	*Fibrobacter*	Bacteria
			357.63	*Veillonellaceae* UCG-001	Bacteria
			352.75	*Anaeroplasma*	Bacteria

## Discussion

This study assessed the microbial groups (bacteria, archaea, fungi and protozoa) in the rumen and identified the age-discriminatory taxa and full maturation of rumen microbiota, keystone species of dynamic intra- and inter- microbial group interactions of natural grazing yaks at varied growth stages from birth to 12 years of age.

First, this study determined that rumen prokaryotes (bacteria and archaea) and eukaryotes (fungi and protozoa) colonized the rumen of natural grazing yaks at different life stages and matured differently. Among the four groups, the bacterial community colonized the rumen before day 7, which is similar to studies on dairy calves. For example, previous studies revealed that bacteria were detected in the rumen of dairy calves as early as birth and for the first week (1–7 days) of age [21, 33, 34], suggesting that rumen bacterial colonization occurs before the intake of solid food. The predominant bacterial phylum, *Bacteroidetes*, was found across all age groups, and the relative abundance of this phylum reached a maximum at 6 months of age, which is similar to the changes in the rumen of dairy cows from birth to 2 years of age [21]. *Prevotella* 1 was the dominant genus (21.7%), and its abundance remained stable after 3 years of age (fed solely with grass). At the early growth stage (days 7 and 14, the main diet was colostrum), the relative abundance of *Bacteroides* was higher than that of *Prevotella* 1 (0.53% vs 0.46 and 4.1% vs 1.9%, respectively). A previous study reported that the genus *Prevotella* (72%) was prominent in older animals (6 months and 2 years of age, fed solely with concentrate), while the genus *Bacteroides* dominated in the rumen of newborns (1–3 days of age, fed solely with colostrum) [21]. In addition, as the diet changed from milk to concentrate, the relative abundance of the genus *Bacteroides* in the rumen of dairy calves decreased from 16.9 to 7.1%, and the relative abundance of the genus *Prevotella* increased to 41.5% with increasing intake of concentrate [20]. However, in the present study, the relative abundance of the genus *Prevotella* 1 varied after grazing solely on natural grass (between 2 and 3 years of age), suggesting that the external living environment of grazing yaks is more variable than that of intensively farmed dairy cows. The dominance of the genus *Prevotella* 1 has also been reported in the rumen of 4-year-old captive yaks fed different doses of slow-release urea [35] as well as yaks (45 ± 5 months) under different feeding regimes [15]. This finding suggests that *Prevotella* 1 may be a conserved ‘core microbiota’ member in the rumen of grazing yaks. Previous studies have determined that members of this genus are involved in hemicellulose, protein [36] and starch degradation [37] but are not regarded as highly cellulolytic bacteria [38]. Future studies are needed to identify whether and how this predominant taxon functions in the rumen of natural grazing yaks raised in the harsh Qinghai-Tibetan Plateau environment.

Archaea were only detected after 14 days of age, which is different from dairy cows. A few studies have reported that archaea colonize the rumen of dairy calves during the first week of life [33, 38, 39], and one study even reported colonization from birth using a qPCR-based approach [40]. These findings suggest that the colonization of archaea in the rumen of natural grazing yaks differs from that of conventionally reared dairy cattle. At the species level, the *Methanobrevibacter ruminantium* clade and *Methanobrevibacter gottschalkii* clade were dominant in the rumen of yaks irrespective of age, suggesting that methanogenesis potentially occurs during the early growth stages as these methanogens are responsible for methane production [39]. In the present study, the relative abundance of the *Methanobrevibacter gottschalkii* clade peaked at 6 months of age and then gradually decreased with age, whereas that of the *Methanobrevibacter ruminantium* clade reached a minimum at 6 months of age and then increased with age, suggesting that these two species may compete in the rumen of grazing yaks during the early growth stages. In addition, these two species were dominant out of 32 ruminant species [41], indicating that these two species may be core methanogens in the rumen of all ruminants.

Fungi were only detected after 1 month of age, which is different from cattle. A previous study reported that fungi were detected in the rumen of dairy calves [33] and lambs [40] at 7 days of age, which is earlier than what we found in yaks. It is noticeable that we only collected rumen samples at 7 days of age, which may not be early enough to determine the exact time when fungi started to inhabit the rumen of grazing yaks. Future studies using the same method to analyze rumen samples collected at birth and confirm the time of initial colonization fungi in the rumen of grazing yaks are necessary. The phylum *Neocallimastigomycota* was predominant in the rumen of grazing yaks irrespective of age, and this result is consistent with findings of the rumen of dairy calves (from 7 days to 63 days) [33] and dairy cows [24]. Similar to a previous finding in dairy calves [33], unidentified *Neocallimastigaceae*, *Caecomyces*, and *Orpinomyces* were dominant in the rumen of grazing yaks irrespective of growth stage, indicating that these taxa may play important roles in rumen development during the early growth stages of ruminants.

Similar to fungi, protozoa were detected in the rumen of yaks after 1 month of age. A previous study detected protozoa in the rumen of lambs at approximately 21 days of age [42], which is earlier than that of yaks. In the present study, grazing yaks drank water from rivers or lakes instead of water troughs, which may preclude the colonization of rumen protozoa, as drinking water has been identified as a main source for rumen protozoa colonization [43]. Furthermore, rumen samples of newborn animals were collected in the winter, and rumen protozoa colonization may also be affected, as environmental temperature has been reported to affect the population and diversity of protozoa from soil and water [44, 45]. At the genus level, *Entodinium* and *Dasytricha* were predominant in the rumen of grazing yaks, which is consistent with previous findings in Yellow [46] and Hanwoo [47] cattle. In addition, a recent study found that the abundance of *Dasytricha* spp. increased, whereas the abundance of *Entodinium* decreased with increasing dietary fiber content [48]. In the present study, the genera *Isotricha* and *Dasytricha* were detected after 3 months of age when the diet of grazing yaks naturally changed from mother milk to native grass. *Isotricha* and *Dasytricha* play very important roles in utilizing soluble sugars and controlling the rate of carbohydrate fermentation [49]. This observation indicates that the selection of protozoa in the rumen of grazing yaks may be driven by dietary fiber contents.

In addition to identifying the microbiota that colonize rumen, it is also important to know when such microbiota becomes established. This provides information for the potential window to manipulate microbiota, as it is more difficult to alter fully established microbiota [50]. We characterized the rumen microbiota in grazing yaks fully matured between 5 and 8 years of age. Specifically, rumen archaea became fully maturated at approximately 5 years old, while the other microbial groups (bacteria, fungi and protozoa) became fully maturated between 5 and 8 years of age, indicating that the time for rumen microbiota to become fully maturated differs among these four microbial groups. We also observed that this maturation pattern differs based on the changes in alpha diversity. Although the alpha diversity indices became stable at 2 years of age, this does not mean that the rumen microbiota become fully mature as the function of some taxa may still change with host physiological development [51]. In addition, it has been reported that malnutrition delays gut microbiota maturation [52]. The grazing yaks in this study are malnourished conditions, as in the long cold season, the Qinghai-Tibetan Plateau provides limited food resources. Thus, rumen microbial maturation in grazing yaks may be delayed. Yak is traditionally considered an adult between 4 and 7 years old [53, 54], which is similar to the predicted maturation time of rumen microbiota in this study. These findings suggest that the stability of rumen microbiota diversity may not be an indicator of microbiota maturation in the rumen of grazing yaks. Moreover, the age-discriminatory taxa within each microbial group were identified, and these taxa have the power to discriminate the age of the rumen microbiota. Among these, *Olsenella* (bacteria)*,* Group 10 sp., belonging to the family *Methanomassiliicoccaceae* (archaea*)*, *Orpinomyces* (fungi) and *Dasytricha* (protozoa) contributed the most to discriminating the age of the rumen microbiota, as their abundance tended to be stable across growth stages, and they were present in most age groups [55]. Therefore, future studies are necessary to identify to what extent the age-discriminatory taxa are indicative of the normal development of the rumen microbiota as well as to determine when biomarkers of rumen microbiota are established.

Similar to previous reports in dairy calves from birth to adulthood [21] and pre-weaning dairy calves [33], we also found that inter-individual animal variation in the bacteria and archaea of rumen decreased with age in grazing yaks. This indicates that high fluctuations of rumen bacterial and archaeal communities may be prevalent in the neonates of ruminant species. Based on PCoA analysis, the community of rumen bacteria and archaea was clearly separated between the young age groups (7 days, 14 days and 1 month of age) and the older age groups (2–12 years of age), which also was corresponded to two feeding regimes: mother’s milk and natural grass. This suggests that both age and diet type contribute to the colonization of bacteria and archaea in the rumen of grazing yaks, as previously reported in dairy calves [25]. However, previous studies on dairy calves [33] and goats [56] have found that rumen archaeal communities are less sensitive to age change, and further studies should be performed to determine which factor contributes more to determining the development of archaea in the rumen of grazing yaks. No clear separation was observed in the protozoal and fungal profiles. The rumen fungal community was also reported to be resistant to changes in age between primiparous and multiparous cows [24], and the rumen protozoal diversity changes little throughout life, although the relative abundance of protozoal species in rumen fluctuates with diet [28]. These results suggest that fungal and protozoal communities may be less sensitive to changes in age and diet type than other microbial groups.

Rumen microbiota work synergistically to perform various metabolic functions that ferment fibrous plant materials. Based on the intra-interactions of rumen bacteria, archaea, fungi and protozoa across the lifetime of yaks, we identified different interactions within each microbial group. In the bacterial network, few strong negative correlations were found; however, few strong positive correlations were found within bacteria in the rumen of other ruminant species [41]. Previous studies have reported that the dynamic interactions of rumen microbiota are specific to diet in dairy cows [57] and can be affected by the host [41]. Furthermore, these interactions differed in the rumen of multiparous cows compared to those in primiparous cows [24], suggesting that the interactions of rumen bacteria could also be affected by age and host physiological state. For the archaeal network, the *Methanobrevibacter gottschalkii* clade and *Methanobrevibacter ruminantium* clade had a negative relationship, which is similar to previous findings in the rumen of adult sheep, cattle and deer fed different diets [58] indicating that these two species compete for H_2_ in the rumen [59]. For the fungal network, the genus *Caecomyces* and the unclassified family *Neocallimastigaceae* had a strong negative correlation, which is different from what has been reported in the rumen of adult cows fed different diets (80% forage and 20% concentrate) [24], suggesting that the interaction between rumen fungi may be affected by host and diet. A significant negative correlation was observed between subclass *Trichostomatia* and *Entodinium*. Members of the subclass *Trichostomatia* have greater endoglucanase and xylanase activity, while members of the genus *Entodinium* have only weak endoglucanase and xylanase activity [60], suggesting that the lack of co-occurrence may be due to the exploitation of different opportunities. A previous study revealed that the complexity of the relationships of rumen microbes shifts from nongrazing to grazing and that these microbes work together to adapt to the dietary shift in sheep [61], which is similar to our results. Based on these findings, it is suggested that specific associations within each microbial group may exist across diets and hosts, with the ability to adapt to the specific life environment and potentially utilize the available substrates.

For the identified inter-interactions among bacteria, archaea, fungi and protozoa under different growth stages; fungi were (*Caecomyces*) positively correlated with bacteria (*Prevotellaceae*), reflecting a synergistic relationship, and the degradation of plant fiber by fungi appears to facilitate more rapid breakdown of forage by fibrolytic bacteria [57]. The lack of strong associations between archaea and protozoa in this study is similar to previous observations in the rumen of multiparous Nordic Red dairy cows [57] and many different ruminant species from different locations [41], suggesting that the associative patterns between archaea and protozoa are less specific and more random in vivo. In addition, no strong associations were found between bacteria and archaea except for the positive association between *Methanosphaera* sp. ISO3-F5 and *Ruminococcaceae* NK4A214 group. This association is unexpected given that rumen bacteria breakdown complex compounds and produce substrates that methanogens use for growth, mainly, hydrogen and methyl-containing compounds [62]. This indicates that the interactions between bacteria and archaea in the rumen are probably host-specific and warrant future studies.

Last, co-occurrence analysis revealed distinct associative patterns of age-specific taxa in different microbial groups. Specifically, the complexity of the bacterial network of the intra-interactions of age-specific taxa fluctuated greatly at early stages of growth (7 days to 2 years old) but remained stable afterwards, and unique keystone species were identified in almost every age group, indicating that the dynamic association patterns between rumen microbial groups changed with growth stages or diet. Regarding the inter-interactions of age-specific taxa, the change in network complexity is similar to that in the intra-interaction, and the majority of keystone species under different growth stages belong to bacteria, indicating that bacteria may play a more central role in rumen biological networks. In addition, the keystone species identified under each age included age specific and core taxa, suggesting they both play important roles in the rumen of yaks at different growth stages.

## Conclusions

This study comprehensively explored the rumen microbiota (including bacteria, archaea, protozoa and fungi) in grazing yaks for the first time. Our results revealed that the alpha diversity of rumen microbiota increases with age and generally remains stable after 2 years of age. The rumen microbial communities underwent multiple changes with growth stage, where bacteria and archaea were more sensitive to changes in age compared to other microbial groups. In addition, bacteria and archaea were observed before 7 days, while protozoa and fungi were detected at approximately 1 month of age, suggesting that rumen prokaryotes generally appeared earlier than eukaryotes in the rumen of grazing yaks. Furthermore, four rumen microbial groups had their own maturation trajectory over the lifetime of yaks, where rumen archaea fully maturated at approximately 5 years of age and the other microbial groups maturated between 5 and 8 years of age. Distinct associative patterns among ruminal microbial groups were observed in each age group, and the dynamic intra- and inter-interactions among four microbial groups changed across the lifetime of yaks. Rumen bacteria play a central role in the rumen biological networks, as most keystone species in all age groups belonged to bacteria (including *Christensenellaceae* R-7 group, *Prevotella* 1, *Ruminococcaceae* UCG-014 and *Lachnospiraceae*). The interactive analysis of this study provides novel insight into elucidating the dynamic intra- and inter- interactions of rumen microbial groups in grazing yaks across a lifetime, providing a solid basis for the manipulation of rumen at different growth stages to improve performance in the harsh Qinghai-Tibetan Plateau ecosystem. One of the limitations of the current study was the lack of rumen fermentation parameters (such as pH, VFA and ammonia) and quantification of microbial abundance by qPCR due to the challenges of collecting enough rumen samples from yaks, especially from the young age groups (from 7 days to 3 months of age). These data, if available, could provide evidence from a functional perspective to reflect rumen development. Regardless, the findings of this study provide fundamental knowledge of the rumen microbiota of grazing yaks during different growth stages. Future studies on the function of the rumen microbiota and its relationship with host development should be explored to identify whether the rumen microbiota and its maturation and interaction can contribute to host growth and productivity.

## Methods

### Animals and sampling

The yaks enrolled in this study were weaned naturally (approximately 12–18 months old) and grazed naturally together year round (without concentrate supplementation) on native pastures from Wushaoling of Tianzhu Autonomous County, Gansu Province (37°12.4′N, 102°51.7′E; altitude of 3154 m). Specifically, all the animals used in this study were from the same herd and they all grazed together in an alpine meadow on the Qinghai-Tibetan Plateau, and they drank water from the local river or the snow meltwater. In addition, animals were grazing on the grassland except at sampling time (animals were rounded up at night before the day of sampling). Rumen fluid samples were collected from yaks ranging from 7 days to 12 years old, and the experimental design is shown in Fig. 1. Briefly, rumen fluid samples were repeatedly sampled from 8 yaks from 7 days to 1 year old. Rumen fluid samples from another 36 yaks aged 2 years (*n* = 6), 3 years (n = 6), 5 years (n = 6), 8 years (n = 6), 10 years (n = 6) and 12 years (n = 6) were collected in 1 day within 3 h. Specifically, rumen samples from yaks aged 6 months to 12 years old were collected via a perforated stainless-steel stomach tube connected to a suction pump prior to morning grazing. The procedure for sample collection from calves (7 days to 4 months, isolated from their mothers on the day before sample collection) was similar except the stainless-steel stomach tube was replaced by a plastic flexible stomach tube. The first 5 ml (from 7 days to 4 months of age) and 20 ml (from 6 months to 12 years of age) rumen fluid were discarded to avoid any contamination with saliva. The rumen fluid (approximately 20–100 ml for yaks aged 7 days to 4 months, 250 ml for yaks aged 6 months to 12 years) was collected and divided into aliquots in 10 ml polypropylene tubes. The rumen samples were immediately deep frozen using liquid nitrogen, transported to the laboratory and stored at − 80 °C prior to DNA extraction.

### DNA extraction

Total genomic DNA was extracted from freeze-dried samples using a PowerSoil DNA Isolation Kit (MoBIO Laboratories, Inc., Carlsbad, CA, USA) according to the manufacturer’s instructions. The concentration and quality of DNA were assessed using a NanoDrop 2000 Spectrophotometer (Thermo Fisher Scientific, Scoresby, Australia) and agarose gel (1.0%) electrophoresis, respectively. The isolated DNA was stored at − 20 °C until downstream analysis.

### Amplification of target genes for rumen microbiota profiling

For rumen microbial profiling, primers Ba9f (GAGTTTGATCMTGGCTCAG) and Ba515Rmod1 (CCGCGGCKGCTGGCAC) targeting the bacterial partial 16S rRNA gene, primers Ar915aF (AGGAATTGGCGGGGGAGCAC) and Ar1386R (GCGGTGTGTGCAAGGAGC) targeting the archaeal partial 16S rRNA gene [41], primers Reg841F (GACTAGGGATTGGAGTGG and Reg1302R (AATTGCAAAGATCTATCCC) targeting the ciliate protozoal 18S rRNA gene [58], and primers MN100F (TCCTACCCTTTGTGAATTTG) and MNGM2 (CTGCGTTCTTCATCGTTGCG) targeting the internal transcribed spacer region of anaerobic fungi [63] were used to generate amplicons for each rumen microbial group. The PCR amplification products were verified using agarose gel (2%) electrophoresis and purified with a Qiagen Gel Extraction Kit (Qiagen, Germany). Mixed samples were prepared by pooling equal amounts of PCR amplicons from each sample and then sequenced on an Illumina PE MiSeq 300 platform to generate 300-bp paired end reads.

### Sequencing data analysis

Among the samples, only 80, 75, 65 and 71 samples were successfully amplified for bacteria, archaea, fungi and protozoa, respectively. Detailed sample numbers for each age group are listed in Table 1. The raw sequence data were assigned to each sample according to the corresponding barcode and were processed using QIIME2 [64]. The DADA2 algorithm [65] as a QIIME2 plugin was used to preprocess the demultiplexed paired-end sequence reads, including quality filtering, denoising, joining paired ends, and removing chimeric sequences. Sequences were clustered into exact sequence variants (ESVs). Next, taxonomy was assigned to ESVs via the “qiime feature-classifier” command using the “classify-sklearn” option [66] against the SILVA 132 database for bacteria and protozoa, the RIM-DB database for archaea, and the UNITE database for fungi. For diversity analyses, sequences were aligned using Mafft [67], and noninformative positions in the alignment were filtered with the ‘mask’ command. Next, a midpoint-rooted phylogenetic tree followed by a phylogenetic tree was performed using the FastTree plugin [68]. Finally, each sample was rarefied to 7158 (bacteria), 3794 (archaea), 4761 (fungi) and 1818 (protozoa) sequences respectively prior to calculating alpha- and beta- diversity metrics; including Faith’s phylogenetic diversity (PD), Chao1 index, and observed OTUs for alpha diversity, and unweighted UniFrac and weighted UniFrac distance for beta diversity.

### Definition of rumen microbiota maturation in grazing yaks using random forests

To identify the characteristics of ESVs of rumen microbiota maturation, random forest regression [31] was used, and the frequency of ESVs in the temporal profiles of rumen microbiota against chronological age with default parameter was regressed in R studio (3.5.3). The model was randomly rebuilt 100 times, and the feature importance scores were averaged across the 100 models [69]. To estimate the minimal number of taxa that generated the lowest cross-validation error needed to predict the final model, the “rfcv” function implemented in the “randomForest” package was applied 100 times. A sparse model was built on a subset of predictive variables that were determined according to their feature importance scores and the number of taxa to be included, which was used to predict chronological age, described as “microbiota age”. A smoothing spline function in R studio (3.5.3) was fit between microbiota age and the corresponding chronological age of the animals (at the time of rumen sample collection). When the curve reached a plateau, the microbiota reached full maturity [69].

### Co-occurrence network analysis

ESVs with relative abundances > 0.01% were used for co-occurrence analysis to explore the co-occurrence patterns within and between bacteria, archaea, fungi and protozoa. Taxa that occurred in > 50% of the population from each age group and were present in all age groups were termed “core taxa” [70, 71]; these taxa were used to explore interactions of rumen microbes from neonates to adults. Meanwhile, the taxa that were only detected in at least 50% of the samples from each age group were regarded as “age-specific microbiota”; these taxa were applied to elucidate hub taxa in different age groups. The co-occurrence network was built based on the Spearman correlation matrix calculated with the WGCNA package [72], and *P*-values for multiple testing were adjusted using the Benjamini and Hochberg false discovery rate (FDR) controlling procedure [73]. The nodes in the network represent rumen taxa, whereas the edges (connections) correspond to the correlation between nodes. The network images were generated using Cytoscape 3.7.1 [74]. When exploring the interactions of core taxa, only correlations with an R-corr absolute value greater than 0.3 and adjusted P-value less than 0.05 were plotted. Regarding the threshold of age-specific taxa, to better reflect the entire microbial relationships, correlations with an R-corr absolute value ≥0.5 were used for further analysis without filtering *P* values. In addition to network topology parameters, betweenness centrality was used to measure the extent to which a node lied on the shortest path between other nodes in the network. Other topological features, such as the degree centrality, transitivity and closeness centrality, were also calculated using the igraph package to describe the complexity of this network. Nodes with the highest betweenness centrality scores were considered keystone species in co-occurrence networks [75].

### Statistical analysis

The alpha diversity indices among different age groups were compared using the nonparametric Kruskal-Wallis testing method to determine if there were significant differences using RStudio (3.5.3). Principal coordinates analysis (PCoA) was performed using the qiime2R package [76] to visualize dissimilarity based on weighted and unweighted UniFrac distance metrics. A pairwise PERMANOVA test was carried out to test the significant differences in microbiome beta diversity among age groups, and default permutations were used to calculate the *P*-value in QIIME2 [77]. In addition, we employed DESeq2 [30] implemented in RStudio to investigate the differentially abundant taxa in different age groups, and this method could handle the uneven sample size during pairwise comparison between groups. The Mann-Whitney U test, which can handle uneven samples [78], was selected to test the alpha diversity indices to compare each group. DESeq2 uses differential expression statistical Wald tests and is adjusted by applying the Benjamini–Hochberg method to correct for multiple hypothesis testing. The FDR cutoff was set at 0.05. Most of the figures presented in this study were generated by RStudio using the corresponding packages unless otherwise noted, and d, m and y indicate day, month and year, respectively, in all tables and figures throughout the manuscript.

## Supplementary information

**Additional file 1: Figure S1** Alpha diversity rarefaction curves of rumen bacteria, archaea, fungi and protozoa.

**Additional file 2 Table S1.** The statistical analysis of alpha diversity from 7 days to 1 year based on repeated methods ANOVA (mixed-effects model). **Table S2.** The results of the pairwise PERMANOVA test. **Table S3.** The results of DESeq2 analysis. **Table S4.** The results of age discriminatory feature importance score. **Table S5.** The topological features of intra-interactions of core taxa in each rumen microbial kingdom. **Table S6.** The topological features of inter-interactions of core taxa among rumen microbial kingdoms. **Table S7.** The keystone species in the network within each age group.

**Additional file 3 Figure S2A, S2B, S2C, S2D** Microbial UniFrac dissimilarity in each rumen microbial group in grazing yaks. Box plot showing within-group similarity, which was calculated using weighted and unweighted UniFrac metrics based on the average pairwise dissimilarity between each paired sample within different groups. A-D indicate bacteria, archaea, fungi and protozoa, respectively.

**Additional file 4 Figure S3A., S3B, S3C, S3D** Taxonomic composition of each ruminal microbial kingdom at the phylum level. Bacteria (A), archaea (B), fungi (C), and protozoa (D).

**Additional file 5 Figure S4A., S4B, S4C, S4D** Heatmap analysis of the relative abundance of rumen microbiota. The relative abundance was log10 transformed, and A-D indicate bacteria, archaea, fungi and protozoa, respectively.

**Additional file 6 Figure S5** Co-occurrence network analysis of inter-interactions between core taxa among microbial kingdoms of rumen. Blue edges correspond to negative correlations, and red edges correspond to positive correlations. The size of the nodes is related to the relative abundance of the taxa. The lines in red and blue denote positive and negative correlations, respectively. The nodes are referred to as keystone species and are highlighted in light purple, and the taxonomic names of keystone species are indicated in the network figures. The taxonomic information of the nodes is shown at the bottom of the figure.

## Data Availability

Raw sequencing data have been deposited at the NCBI Sequence Read Archive (SRA) under BioProject ID PRJNA566442.

## Abbreviations

ESVs: Exact sequence variants
16S rRNA: 16 Svedberg ribosomal ribonucleic acid
18S rRNA: 18 Svedberg ribosomal ribonucleic acid
PCR: Polymerase chain reaction
PCoA: Principal coordinate analysis
FDR: False discovery rate
