# Supplementary figures and images for: Survey of rumen microbiota of domestic grazing yak during different growth stages revealed novel maturation patterns of four key microbial groups and their dynamic interactions
Source: Anim Microbiome. 2020 Jul 14;2:23. doi: 10.1186/s42523-020-00042-8 (PMC7807461; doi:10.1186/s42523-020-00042-8)

Observed\_otu

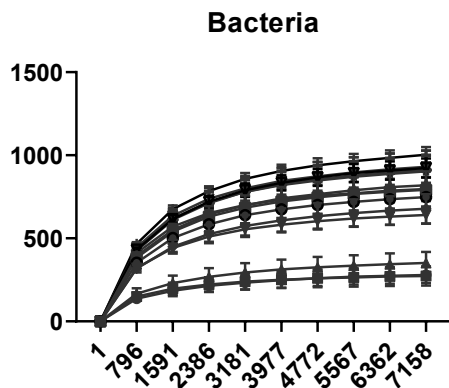

7d  
14d  
1m  
2m  
3m  
4m  
6m  
1y  
2y  
3y  
5y  
8y  
10y  
12y

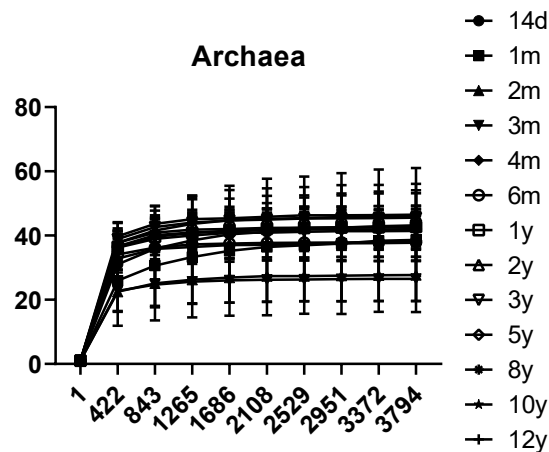

14d  
1m  
2m  
3m  
4m  
6m  
1y  
2y  
3y  
5y  
8y  
10y  
12y

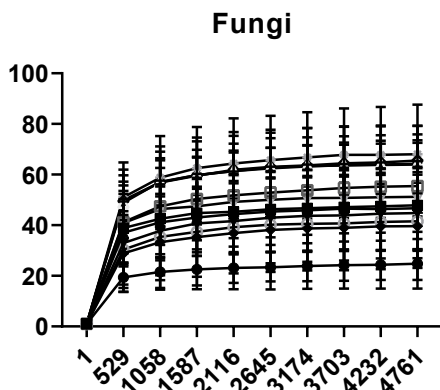

1m  
2m  
3m  
4m  
6m  
1y  
2y  
3y  
5y  
8y  
10y  
12y

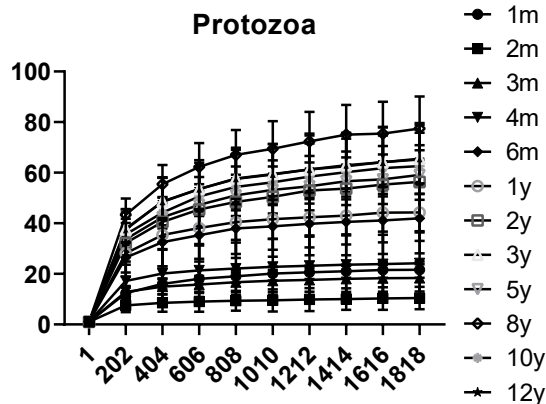

1m  
2m  
3m  
4m  
6m  
1y  
2y  
3y  
5y  
8y  
10y  
12y

Sequencing depth

Supplement: Supplementary file 1 — Additional file 1: Figure S1 Alpha diversity rarefaction curves of rumen bacteria, archaea, fungi and protozoa. [file 42523_2020_42_MOESM1_ESM.pdf]

A

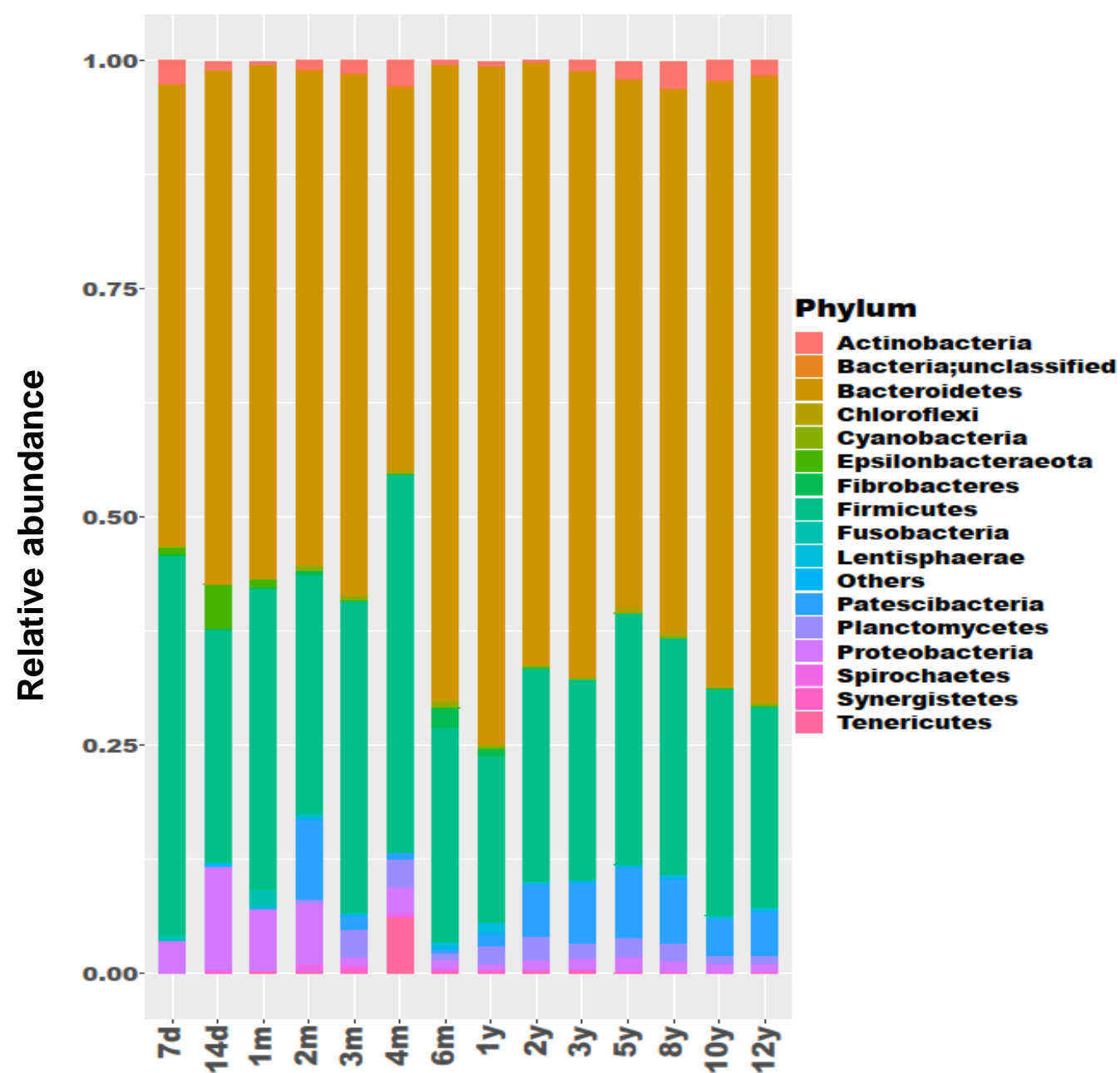

B

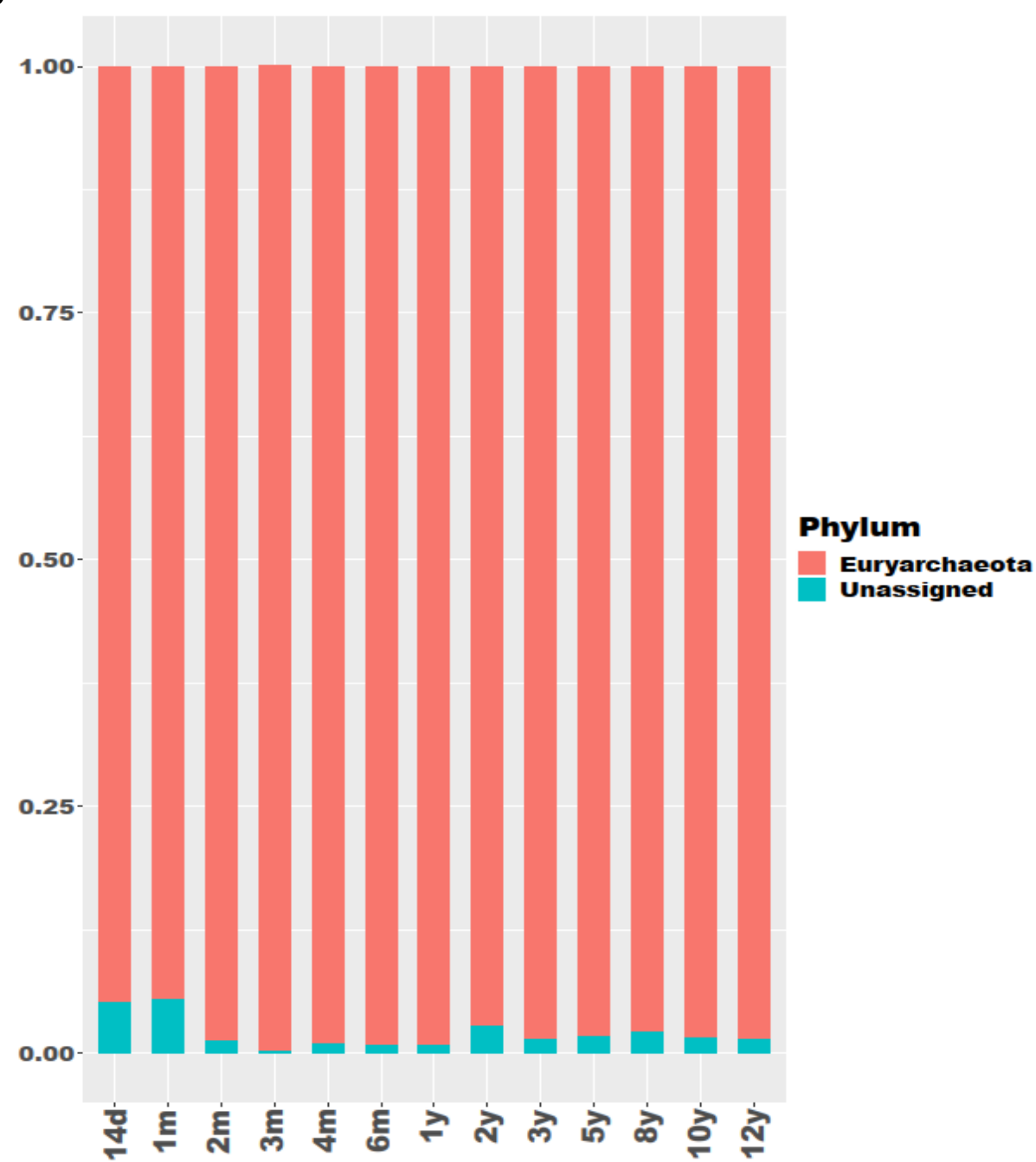

C

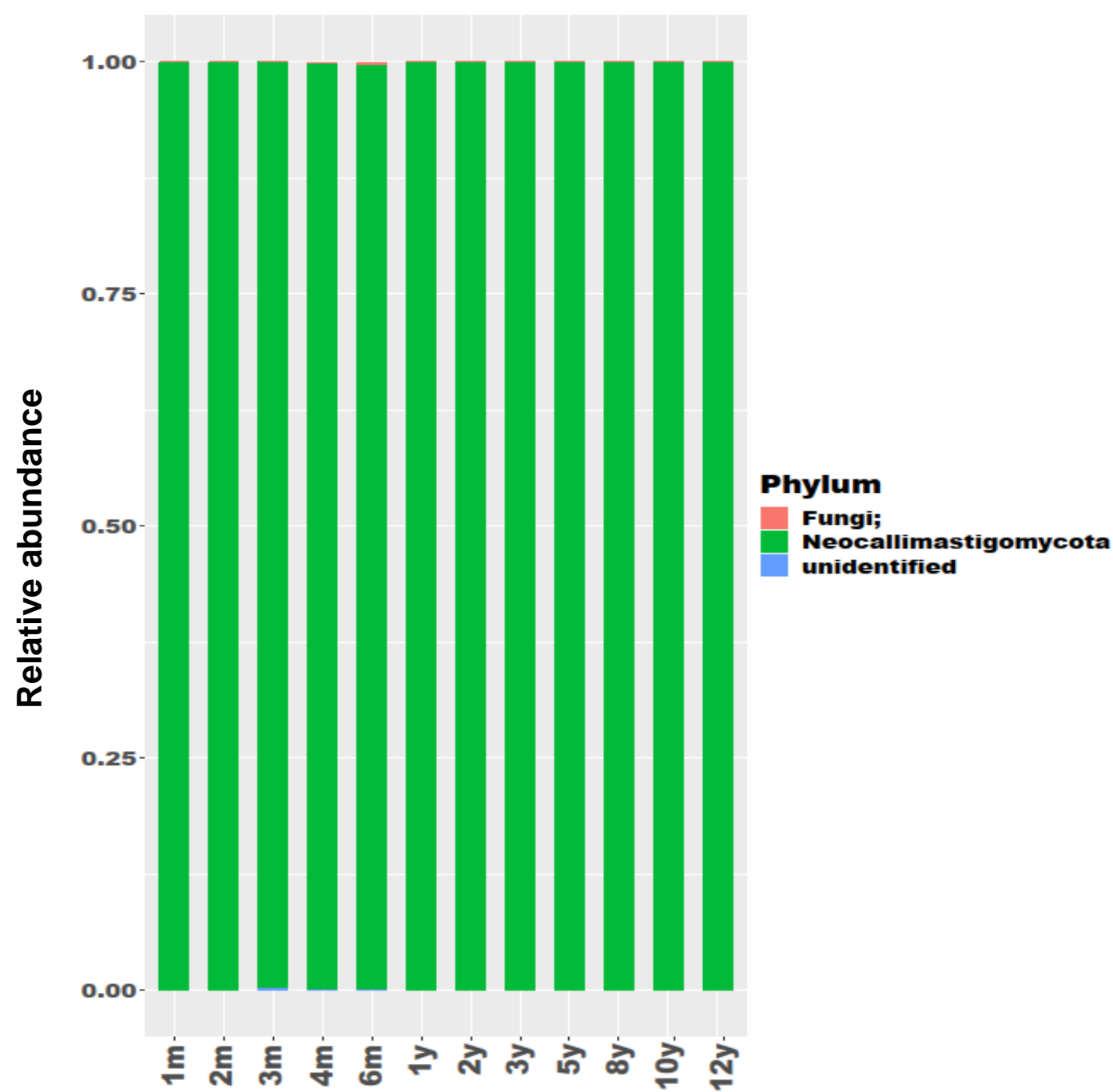

D

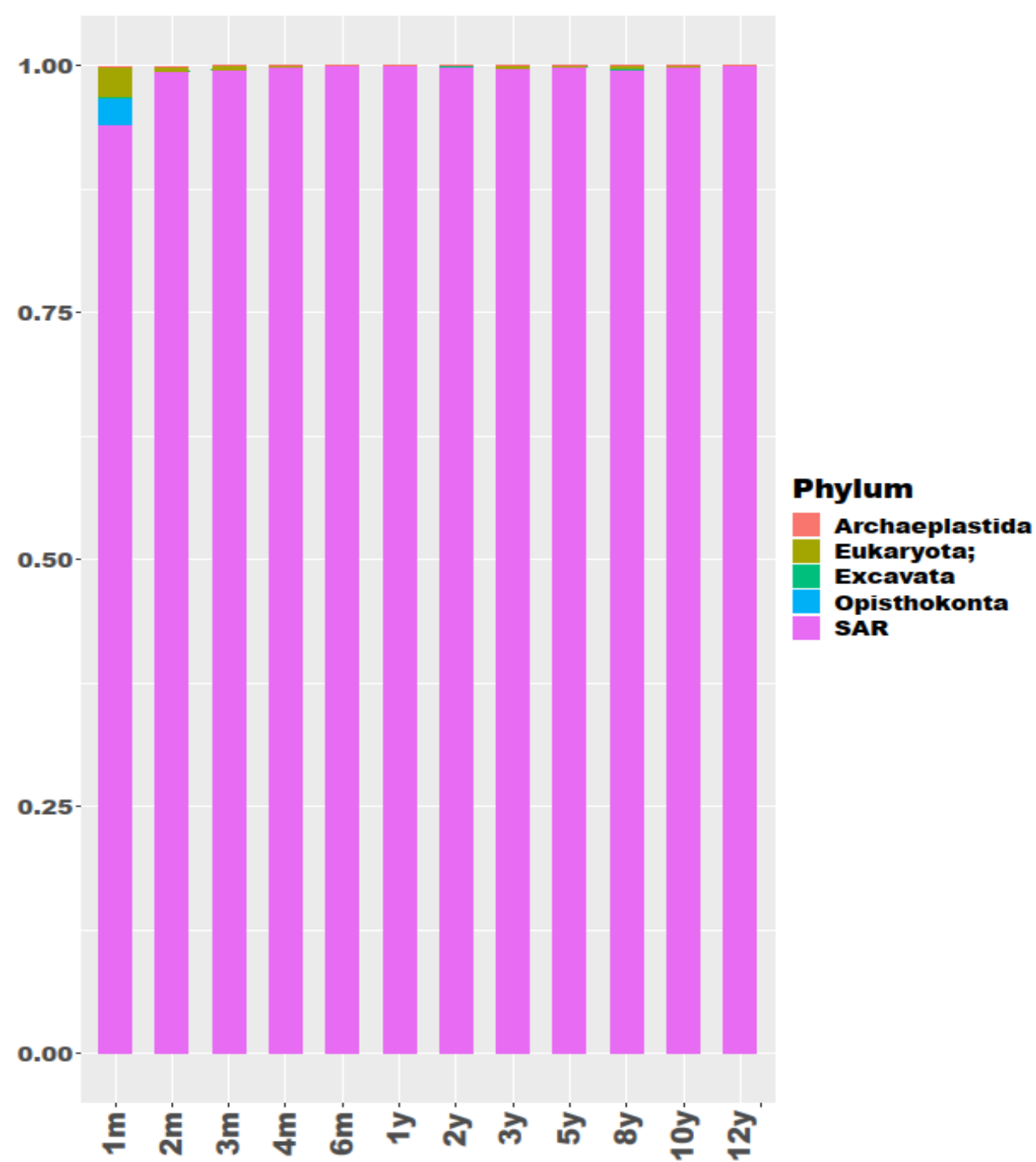

Supplement: Supplementary file 3 — Additional file 3 Figure S2A, S2B, S2C, S2D Microbial UniFrac dissimilarity in each rumen microbial group in grazing yaks. Box plot showing within-group similarity, which was calculated using weighted and unweighted UniFrac metrics based on the average pairwise dissimilarity between each paired sample within different groups. A-D indicate bacteria, archaea, fungi and protozoa, respectively. [file 42523_2020_42_MOESM3_ESM.pdf]

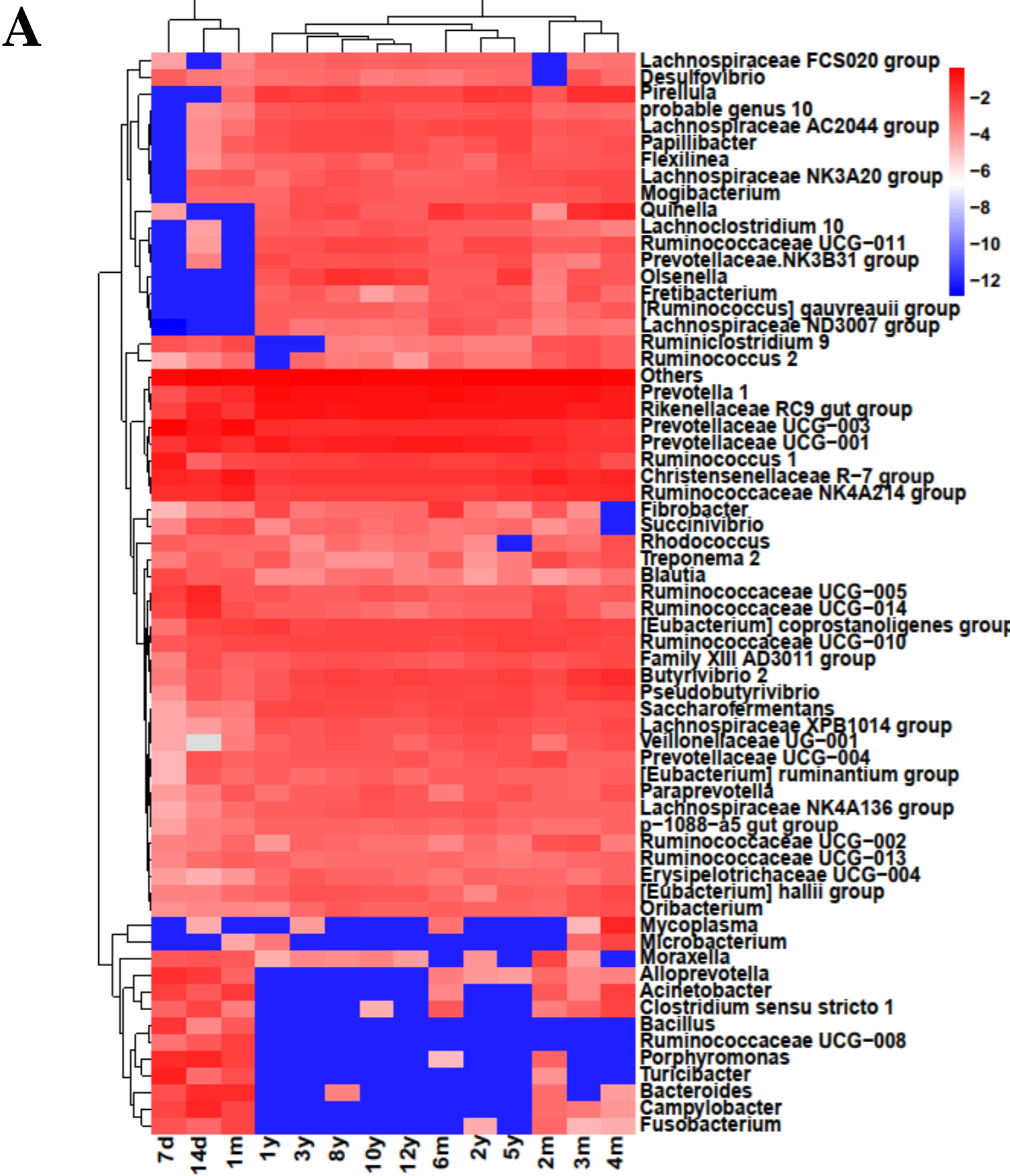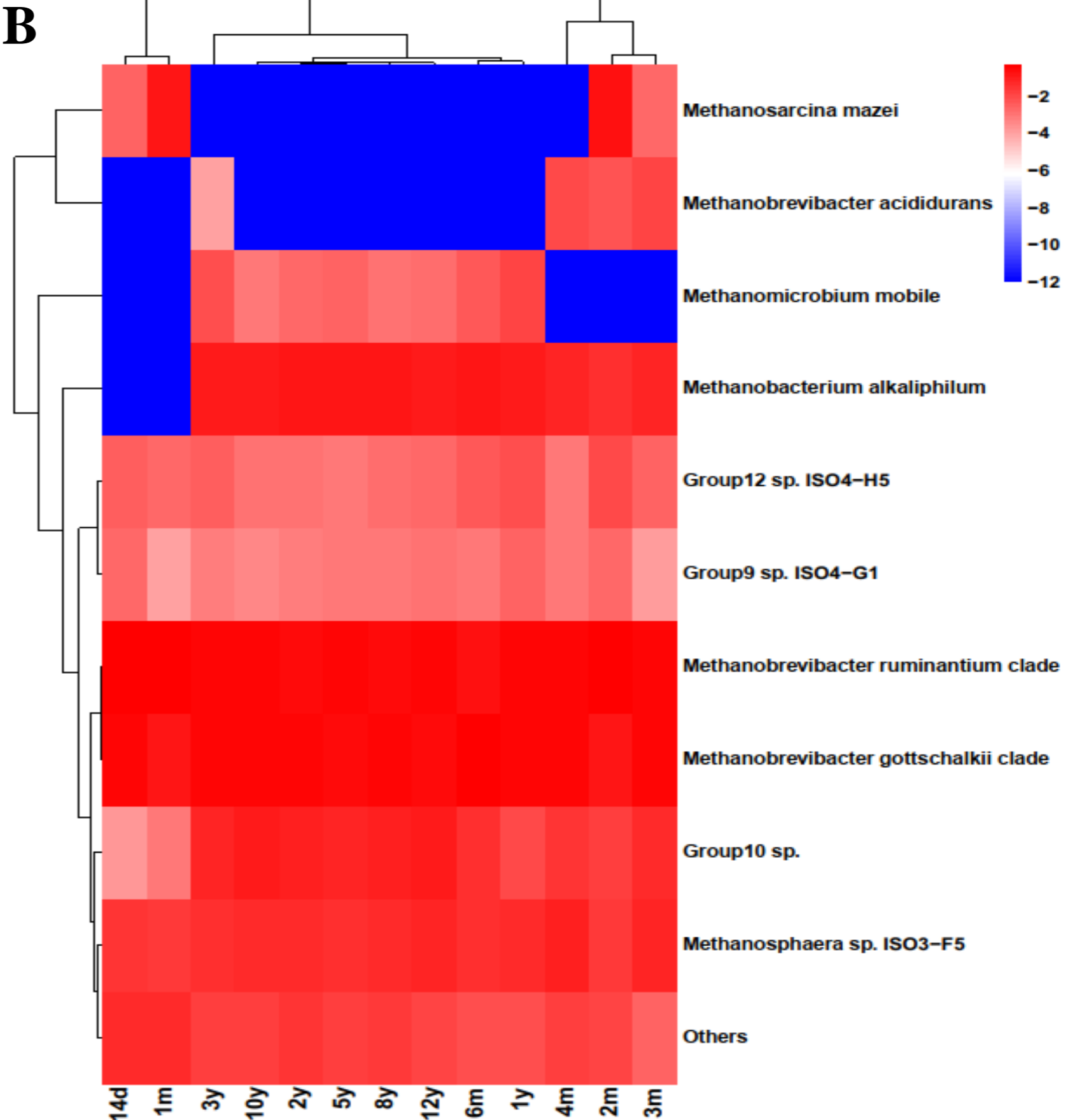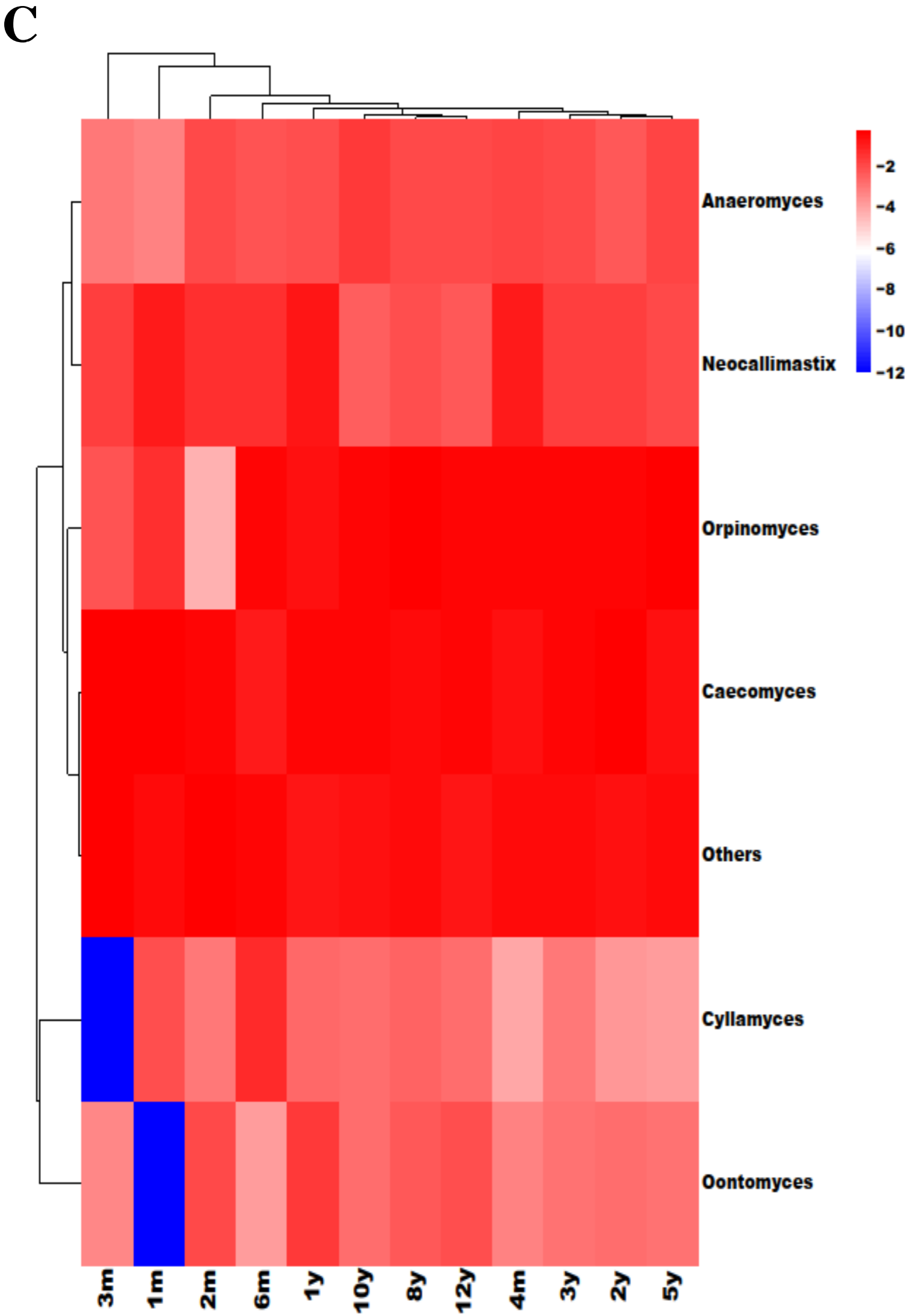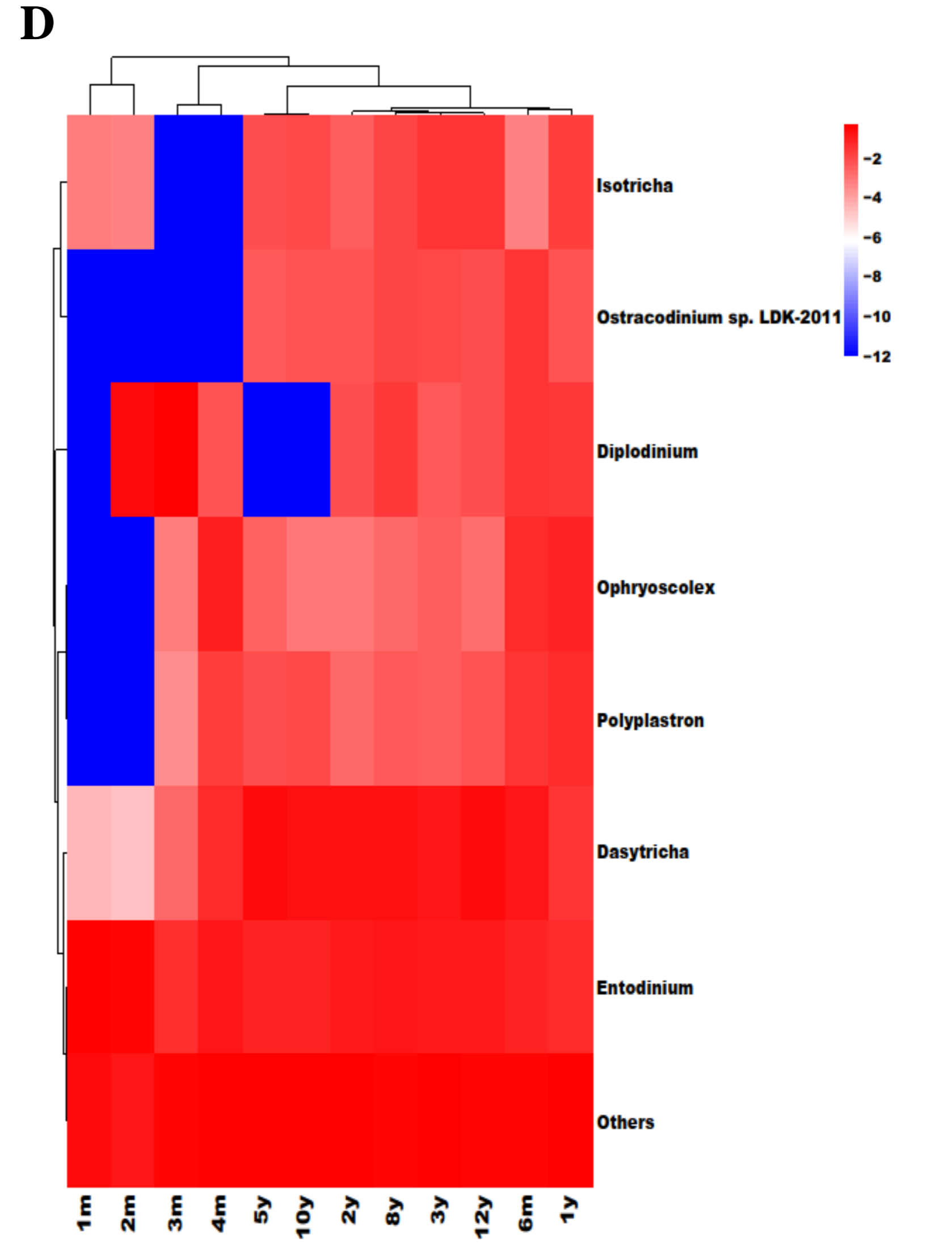

Supplement: Supplementary file 4 — Additional file 4 Figure S3A., S3B, S3C, S3D Taxonomic composition of each ruminal microbial kingdom at the phylum level. Bacteria (A), archaea (B), fungi (C), and protozoa (D). [file 42523_2020_42_MOESM4_ESM.pdf]

A

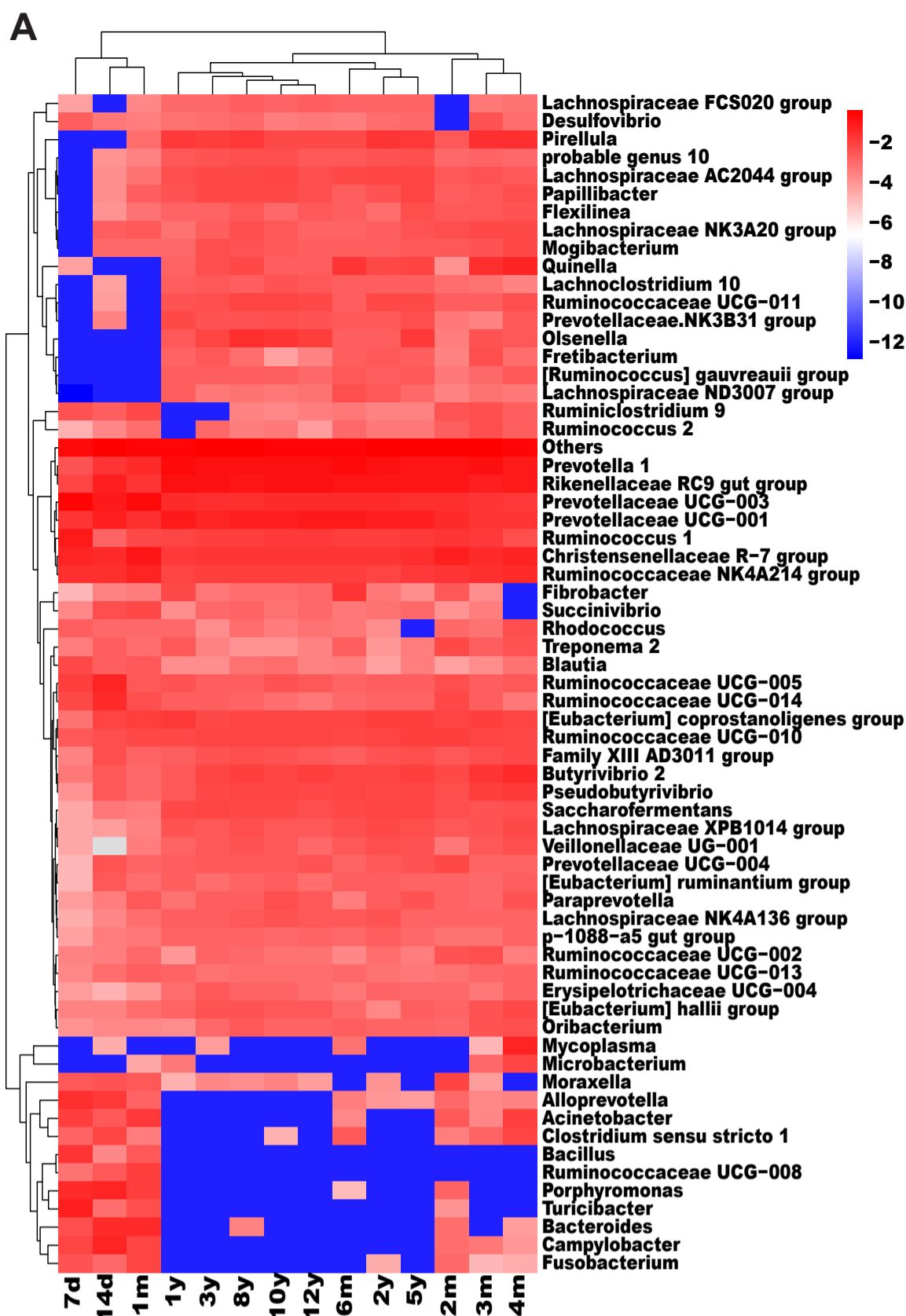

B

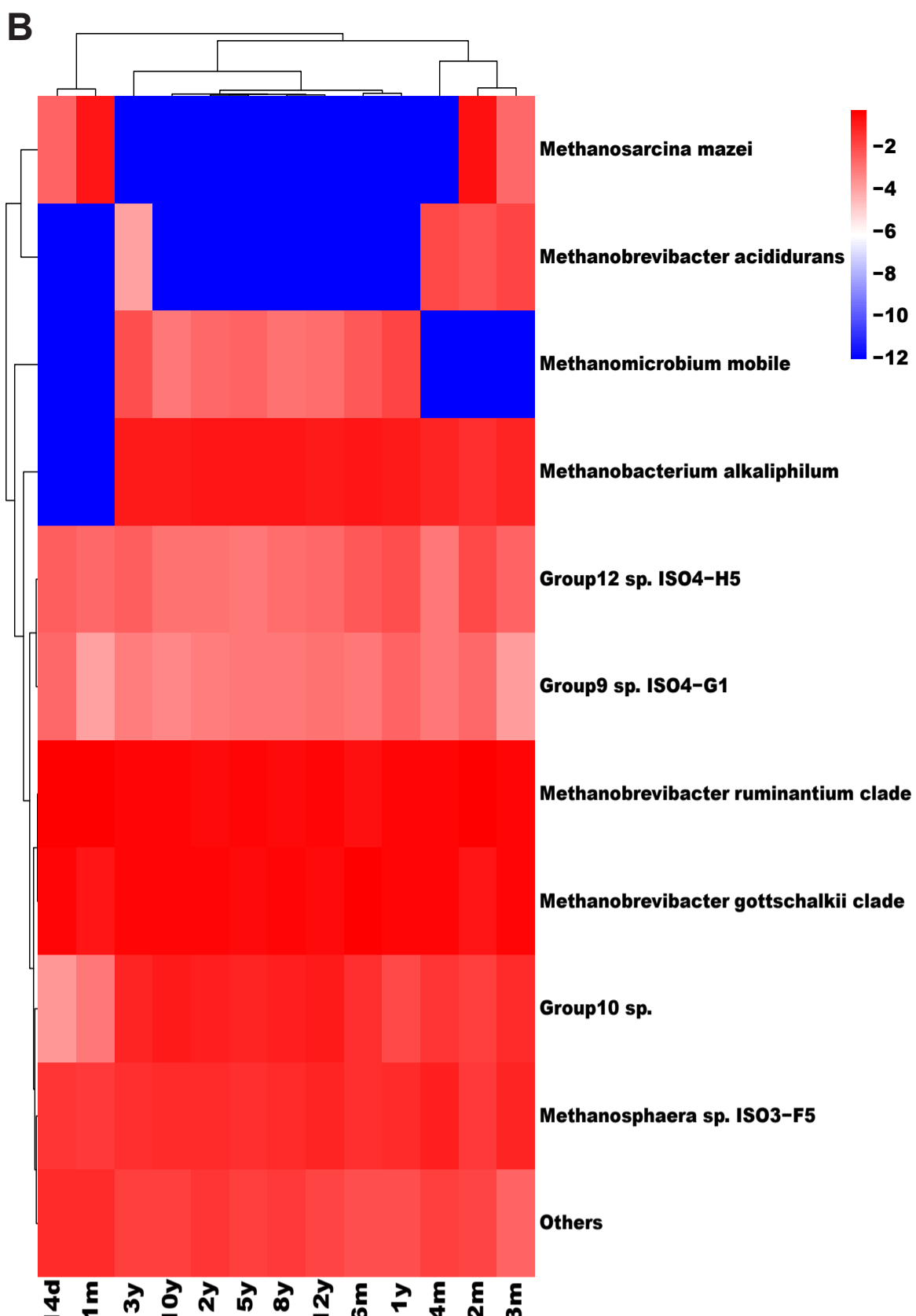

C

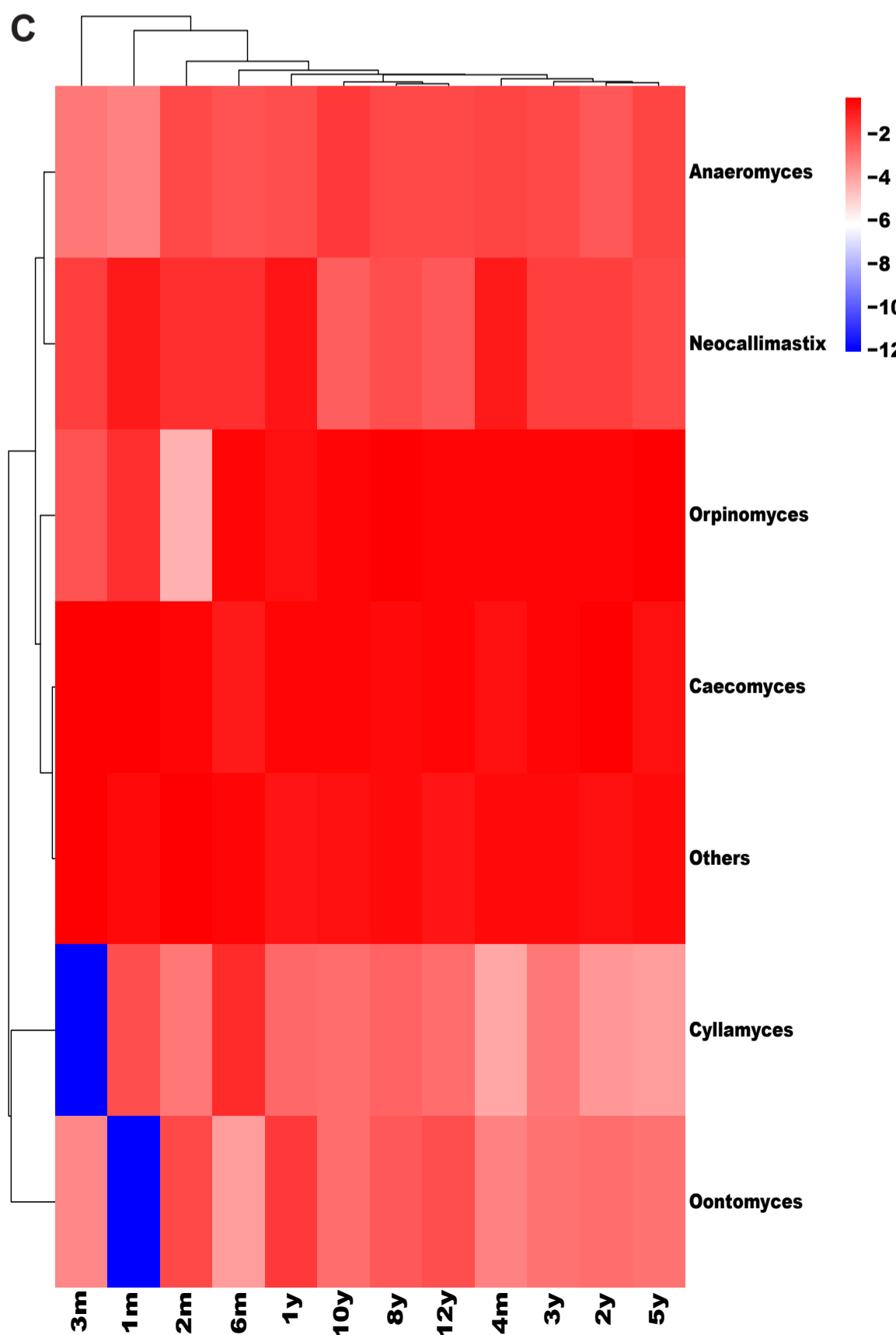

D

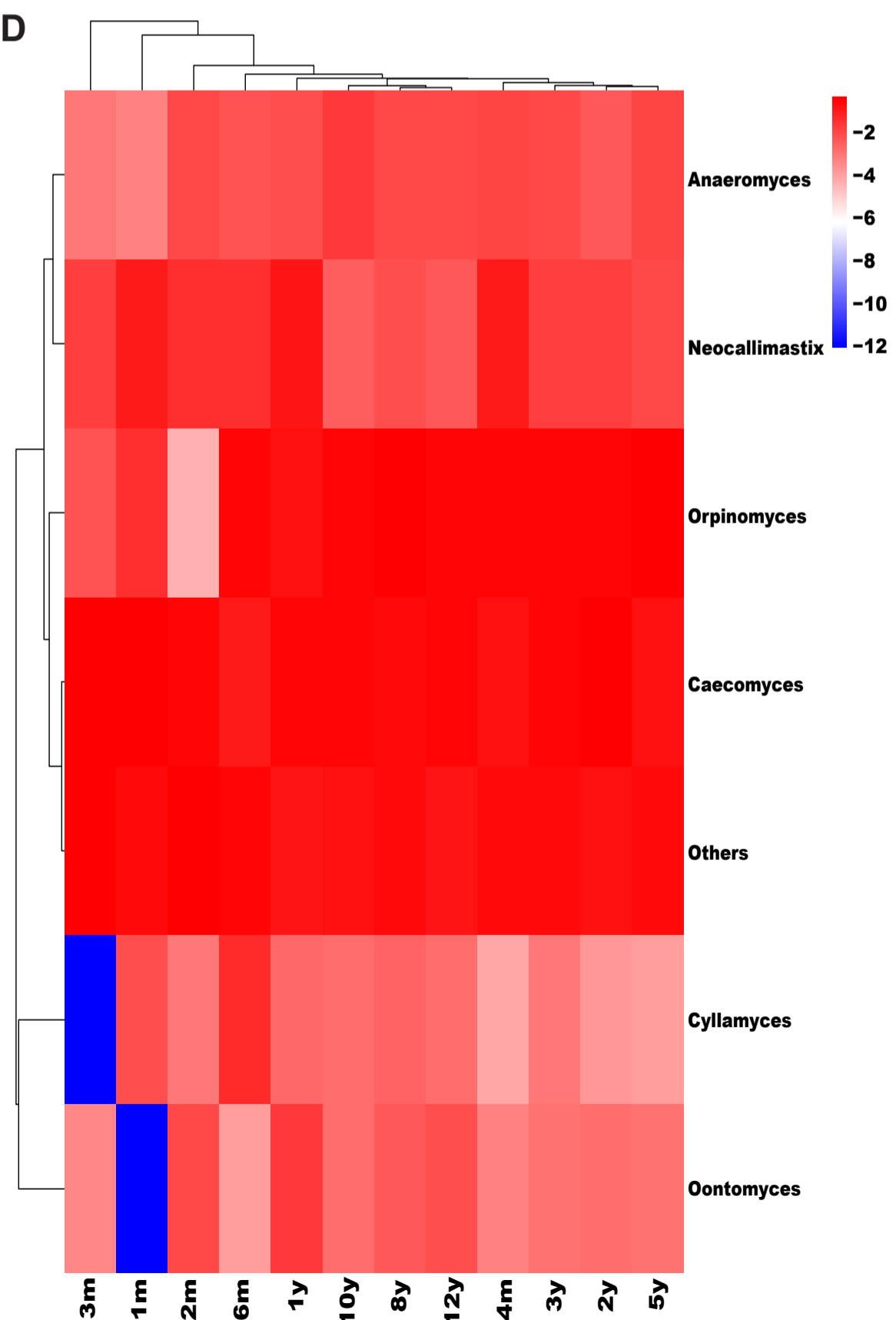

Supplement: Supplementary file 5 — Additional file 5 Figure S4A., S4B, S4C, S4D Heatmap analysis of the relative abundance of rumen microbiota. The relative abundance was log10 transformed, and A-D indicate bacteria, archaea, fungi and protozoa, respectively. [file 42523_2020_42_MOESM5_ESM.pdf]
