# Supplementary material for: Survey of rumen microbiota of domestic grazing yak during different growth stages revealed novel maturation patterns of four key microbial groups and their dynamic interactions
Source: Anim Microbiome. 2020 Jul 14;2:23. doi: 10.1186/s42523-020-00042-8 (PMC7807461; doi:10.1186/s42523-020-00042-8)

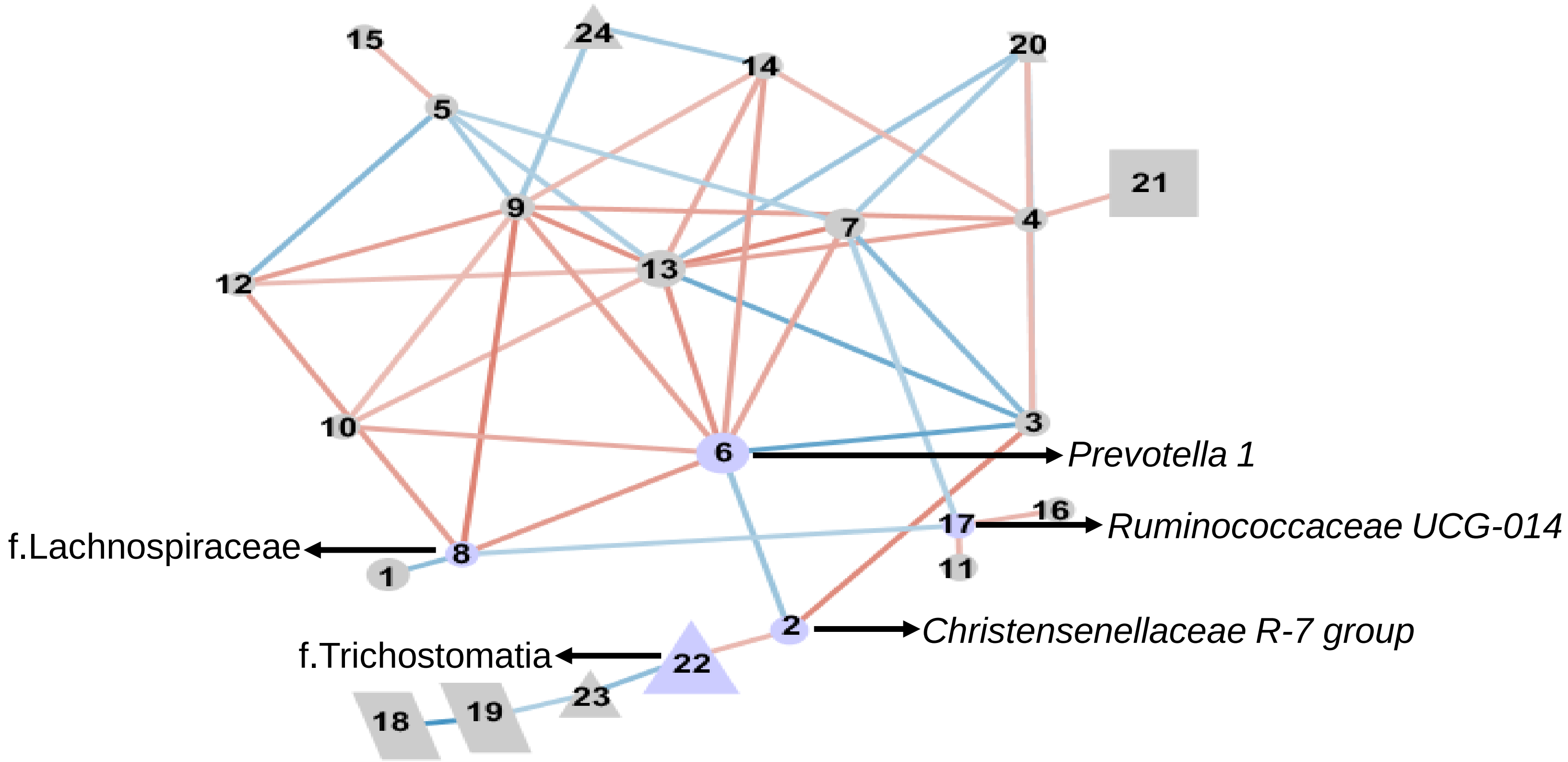

Legend:

1. *Prevotellaceae UCG-003*
2. *Christensenellaceae R-7 group*
3. *Ruminococcaceae NK4A214 group*
4. f.Prevotellaceae
5. f.Ruminococcaceae
6. *Prevotella 1*
7. *Prevotellaceae UCG-001*
8. f.Lachnospiraceae
9. o.Clostridiales
10. *[Eubacterium] coprostanoligenes group*
11. f.F082
12. k.Bacteria
13. *Rikenellaceae RC9 gut group*
14. *Ruminococcus 1*
15. f.Ruminococcaceae
16. *Ruminococcaceae UCG-005*
17. *Ruminococcaceae UCG-014*
18. *Methanobrevibacter ruminantium clade*
19. *Methanobrevibacter gottschalkii clade*
20. *Methanosphaera sp. ISO3-F5*
21. *Caecomyces*
22. f.Trichostomatia
23. *Entodinium*
24. *Dasytricha*

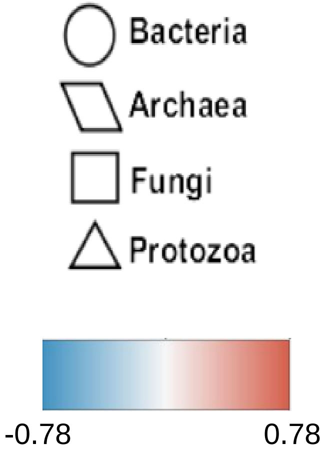

Supplement: Supplementary file 6 — Additional file 6 Figure S5 Co-occurrence network analysis of inter-interactions between core taxa among microbial kingdoms of rumen. Blue edges correspond to negative correlations, and red edges correspond to positive correlations. The size of the nodes is related to the relative abundance of the taxa. The lines in red and blue denote positive and negative correlations, respectively. The nodes are referred to as keystone species and are highlighted in light purple, and the taxonomic names of keystone species are indicated in the network figures. The taxonomic information of the nodes is shown at the bottom of the figure. [file 42523_2020_42_MOESM6_ESM.pdf]
